# Dissection of the Octoploid Strawberry Genome by Deep Sequencing of the Genomes of *Fragaria* Species

**DOI:** 10.1093/dnares/dst049

**Published:** 2013-11-26

**Authors:** Hideki Hirakawa, Kenta Shirasawa, Shunichi Kosugi, Kosuke Tashiro, Shinobu Nakayama, Manabu Yamada, Mistuyo Kohara, Akiko Watanabe, Yoshie Kishida, Tsunakazu Fujishiro, Hisano Tsuruoka, Chiharu Minami, Shigemi Sasamoto, Midori Kato, Keiko Nanri, Akiko Komaki, Tomohiro Yanagi, Qin Guoxin, Fumi Maeda, Masami Ishikawa, Satoru Kuhara, Shusei Sato, Satoshi Tabata, Sachiko N. Isobe

**Affiliations:** 1 Kazusa DNA Research Institute, Kazusa-Kamatari 2-6-7, Kisarazu, Chiba 292-0818, Japan; 2 Department of Bioscience and Biotechnology, Faculty of Agriculture, Kyushu University, Higashi-ku, Fukuoka 812-8581, Japan; 3 Faculty of Agriculture, Kagawa University, 2393 Ikenobe, Miki, Kita, Kagawa 761-0795, Japan; 4 Chongqing Nanshan Botanical Garden, No. 101, Nanshan Garden Road, Nan'an District, Chongqing 400065, China; 5 Chiba Prefectural Agriculture and Forestry Research Center, Crop Breeding Institute, Chousei, Daizenno-Cyou 808, Midori, Chiba 299-4335, Japan; 6 Graduate School of Life Sciences, Tohoku University, Katahira 2-1-1, Aoba, Sendai, Miyagi 980-8577, Japan

**Keywords:** *Fragaria*x *ananassa*, wild *Fragaria* species, genome sequence assembly, comparative analysis, polyploidy

## Abstract

Cultivated strawberry (*Fragaria* x *ananassa*) is octoploid and shows allogamous behaviour. The present study aims at dissecting this octoploid genome through comparison with its wild relatives, *F. iinumae*, *F. nipponica*, *F. nubicola*, and *F. orientalis* by *de novo* whole-genome sequencing on an Illumina and Roche 454 platforms. The total length of the assembled Illumina genome sequences obtained was 698 Mb for *F.* x *ananassa*, and ∼200 Mb each for the four wild species. Subsequently, a virtual reference genome termed FANhybrid_r1.2 was constructed by integrating the sequences of the four homoeologous subgenomes of *F*. x *ananassa*, from which heterozygous regions in the Roche 454 and Illumina genome sequences were eliminated. The total length of FANhybrid_r1.2 thus created was 173.2 Mb with the N50 length of 5137 bp. The Illumina-assembled genome sequences of *F*. x *ananassa* and the four wild species were then mapped onto the reference genome, along with the previously published *F. vesca* genome sequence to establish the subgenomic structure of *F.* x *ananassa*. The strategy adopted in this study has turned out to be successful in dissecting the genome of octoploid *F*. x *ananassa* and appears promising when applied to the analysis of other polyploid plant species.

## 1. Introduction

The cultivated strawberry (*Fragaria* x *ananassa*) is a globally consumed crop species that is grown around the world; in the USA (37.5%), Europe (36.7%), Asia (16.3%), Africa (8.6%), and Oceania (0.8%).^1^ *Fragaria* x *ananassa* is an octoploid species (2*n* = 8*×* = 56) with an estimated genome size of 1C = 708–720 Mb.^2,3^ In addition to its polyploidy, allogamous behaviour in *F.* x *ananassa* contributes further complexity to the genome structure*.* Until now, three genome composition models, namely AABBBBCC,^4^ AAA′A′BBBB,^5^ and AAA′A′BBB′B′,^6^ have been proposed based on cytological and genetic evidence. Of the three models, the most recently proposed model, AAA′A′BBB′B′,^6^ is considered the most probable candidate, since several studies have reported the disomic inheritance of large numbers of DNA markers,^7–9^ which suggests an allopolyploid genome composition in *F.*x *ananassa*.

The genus *Fragaria* belongs to the family Rosaceae and is comprised of one cultivated (*F.*x *ananassa*) and 24 wild species, including 13 diploids, 5 tetraploids, 1 hexaploid, 4 octoploids, and 1 decaploid.^10,11^ The geographic origins of the wild species are distributed throughout Eurasia, North and South America, and Japan. *Fragaria* x *ananassa* originated in the 1700s from a natural hybridization between two octoploids, *F. virginiana* and *F. chiloensis*.^12^ However, the history of evolution from diploid to octoploid species in the genus *Fragaria* remains controversial. Davis *et al.*^3^ proposed that *F. vesca*, *F. nubicola*, and *F. orientalis* were possible progenitors to octoploids, based on the polygenic analysis of internal transcribed spacer sequences of rDNA and chloroplast DNA performed by Potter *et al.*^13^ Rousseau-Gueutin *et al.*^14^ hypothesized that the evolutionary history was based on five subgenomic entries, X1, X2, Y1, Y2, and Z, classified according to two nuclear genes, *GBSSI-2* and *DHAR*. They proposed that the genomes of wild octoploids consisted of Y1′Y1′Y1″Y1″ZZZZ or Y1Y1Y1Y1ZZZZ genomes. The Y1 genomes were considered to be derived from two diploid species, *F. vesca* or *F. mandshurica*, via a tetraploid, *F. orientalis*, whereas *F. iinumae* was presumed to be the progenitor of the Z genome.

*Fragaria vesca*, which is the most plausible progenitor of *F.*x *ananassa*, was selected as the genomic reference for *Fragaria*. Using a fourth-generation inbred line and the Roche 454, Illumina Solexa, and Life Technologies SOLiD platforms, the whole-genome sequence of *F. vesca* was published in 2011.^15^ According to the report, a total of 209.8 Mb were assembled into 272 scaffolds, and 34 809 candidate genes were identified by gene prediction. Later, the original v1.0 pseudomolecule assembly was updated to v1.1.^16^

The *F. vesca* genome sequences have contributed greatly to advances in molecular genetic analysis in *F.* x *ananassa*^8,9^ and are expected to assist in the identification of genes related to agriculturally important traits in *F.* x *ananassa*, such as flowering time,^17^ male sterility,^18^ and stress resistance.^19^ However, consultation of the *F. vesca* genome has limitations, particularly for *F.* x *ananassa*-specific genome regions or genome regions derived from other progenitors. Therefore, whole-genome sequencing of *F.* x *ananassa* is needed for a comprehensive understanding of the genome structure of the species, in parallel with reference to the *F. vesca* genome.

Next-generation sequencing (NGS) technologies have revolutionized *de novo* whole-genome sequencing in most species. This is especially true in plant species, for which genomes are relatively large and complex in structure compared with non-plant genomes.^20,21^ Up to now, whole-genome sequences have been published in more than 50 plant species, including five species in Rosaceae: *F. vesca*,^15^ apple,^22^ pear,^23^ peach,^24^ and Chinese plum.^25^ However, to our knowledge, no reports of *de novo* whole-genome sequencing have been published for any polyploid species. As with strawberry, diploid progenitor species have been employed to advance the genomic understanding of other polyploid crops, such as potato,^26^ cotton,^27^ and banana.^28^ Genome sequencing of polyploid species has been avoided, because the homoeology of subgenomes made sequence assembly difficult. To overcome this difficulty, we attempted to construct a virtual ‘reference genome’ in octoploid strawberry, *F.* x *ananassa*, as a first step for whole-genome sequencing of the species.

In this study, *de novo* whole-genome sequencing was performed in *F.* x *ananassa* using the Illumina (Illumina, Inc., CA, USA) and Roche 454 sequencing platforms (Roche Diagnostics, IN, USA). A virtual reference genome, which integrated genome sequences of homoeologous chromosomes, was constructed by eliminating heterozygous bases in the process of sequence assembly. In a previous study, macrosynteny at the chromosome level was observed between the linkage groups (LGs) in an *F.*x *ananassa* genetic map and the genome of *F. vesca.*^9^ Therefore, we aligned the generated *F.*x *ananassa* scaffolds with the pseudomolecules of *F. vesca*.^15^ In parallel, four wild *Fragaria* species, representing genetic diversity in the genus *Fragaria*, were selected based on simple sequence repeat (SSR) markers and were subjected to whole-genome sequencing using an Illumina platform. The assembled sequences of the wild species, along with the heterozygous *F.* x *ananassa* sequences, were mapped onto the *F.* x *ananassa* reference genome and used to elucidate the genome structures of the *Fragaria* species.

## 2. Materials and methods

### 2.1. Plant materials

A Japanese variety, ‘Reikou’, and its progeny (S_1_) were subjected to genome sequencing as representatives of *F.* x *ananassa.* ‘Reikou’ was bred in the Chiba Prefectural Agriculture and Forestry Research Center. Like other strawberry varieties, ‘Reikou’ maintains heterozygosity in the genome. The S_1_ progenies were sequenced for SNP discovery within ‘Reikou’ for further analysis (data were not shown in this study). A total of 20 wild *Fragaria* species with a diverse range of polyploidy were used in phylogenetic analyses with SSR markers (Supplementary Table S1). Along with the 20 wild species, the following four Japanese *F.* x *ananassa* varieties were subjected to the phylogenic analysis: ‘Reikou’, ‘Hokowase’, ‘Sachinoka’, and ‘Akihime’. Whole-genome sequencing was subsequently performed for four wild species, *F. iinumae*, *F. nipponica*, *F. nubicola*, and *F. orientalis*. The genomic DNA was extracted from young leaves using a DNeasy Plant Mini Kit (Qiagen, Inc., CA, USA). DNA quantification and quality checks were performed using a Nanodrop ND1000 spectrophotometer (Nanodrop Technologies, DE, USA) and 0.8% agarose gel electrophoresis, respectively.

### 2.2. Phylogenetic analysis

Genomic polymorphisms from 24 *Fragaria* accessions (listed in Supplementary Table S1) were analysed using 632 *F.* x *ananassa* and *F. vesca* expressed sequence tag derived SSR markers that randomly mapped onto the 28 LGs of the consensus genetic map of *F.* x *ananassa*^9^ (Supplementary Table S2). PCR was performed as described in Isobe *et al.*^9^ The PCR products were separated using an ABI 3730xl fluorescent fragment analyser (Applied Biosystems, CA, USA). Polymorphisms were investigated using the GeneMapper software (Applied Biosystems). The genetic distances and Jaccard's similarity coefficients of all combinations of any two samples were calculated from the genotypic data using the GGT2 software.^29^ The hierarchical cluster analysis with the multiscale bootstrap was performed for 1000 replications with Ward's method by pvclust package^30^ for the R software (http://www.r-project.org/).

### 2.3. Genome sequencing

**Figure 1.**
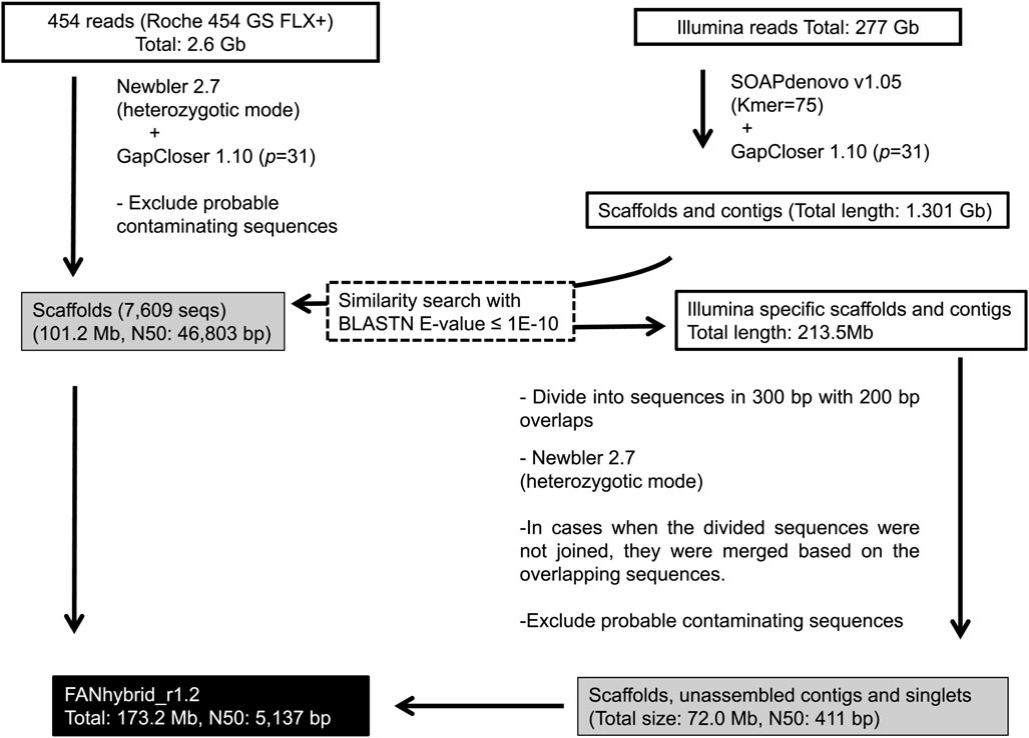
The strategy and status of sequencing and assembly of the reference genome of *F.* x *ananassa* (FANhybrid_r1.2). PE, SE, and MP represent PE, SE, and MP reads, respectively. The FANhybrid_r1.2 sequences (a black box) are consisted with the 454 scaffolds gap-closed with Illumina reads and Illumina-specific assembled sequences (gray boxes).

Whole-genome shotgun sequencing was carried out using the Roche 454 GS FLX+ and Illumina GAIIx/Hiseq 1000 platforms (Roche Diagnostics; Illumina, Inc., Fig. 1, Supplementary Table S3). Total cellular DNA of the ‘Reikou’ variety of *F.*x *ananassa* was used for the construction of single-end (SE) and paired-end (PE) libraries for the Roche 454 sequencing platform according to the instructions provided by the manufacturer. Insert sizes of the PE libraries were 3, 5, 8, and 20 kb.

**Figure 2.**
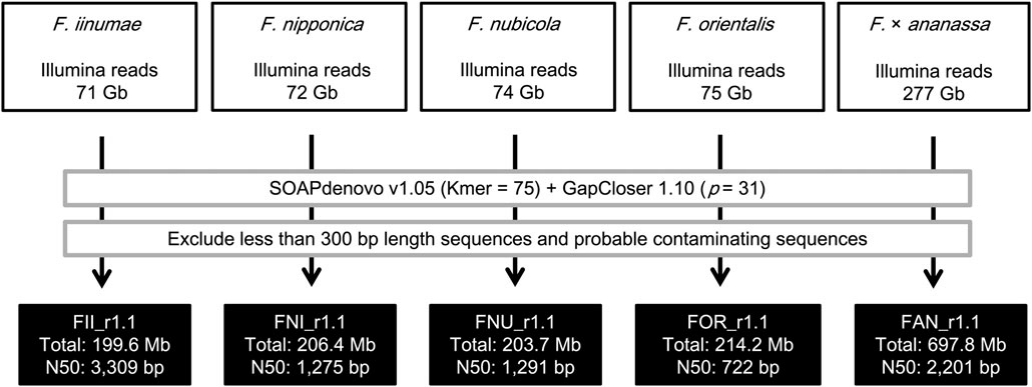
The strategy and status of Illumina sequence assembly of the genomes of *F.*x *ananassa* (FAN_r1.1) and four wild species, *F. iinumae* (FII_r1.1), *F. nipponica* (FNI_r1.1), *F. nubicola* (FNU_r1.1), and *F. orientalis* (FOR_r1.1), based on Illumina reads.

Illumina PE and mate-pair (MP) libraries were constructed from total cellular DNAs of ‘Reikou’, five S_1_ progenies of ‘Reikou’, and four wild species, *F. iinumae*, *F. nipponica*, *F. nubicola*, and *F. orientalis*, according to the instructions provided by the manufacturer (Fig. 2 and Supplementary Table S3). The expected insert sizes and read lengths of the libraries ranged from 290 bp to 2 kb, and 51–101 bp, respectively. Of the developed Illumina libraries, two ‘Reikou’ PE libraries (with insertion sizes of 600 and 400 bp) were subjected to genome sequencing by the Illumina GAIIx, whereas other Illumina libraries were analysed by the Illumina HiSeq 1000. The mixed sequences generated from the five S_1_ progenies were regarded as equivalent to the parental ‘Reikou’ genome and were assembled together with sequence data from the ‘Reikou’ libraries.

### 2.4. Quality control of Illumina reads

The Illumina reads were preprocessed for quality control using the FastX-toolkit, ver. 0.0.13 (http://hannonlab.cshl.edu/fastx_toolkit/). After quality filtering, the sequences that included an N or were trimmed according to the following criteria were excluded from further analysis: (i) bases with quality value <10; (ii) probable artefact reads; and (iii) adaptor sequences consisting of more than five bases at the 3′ terminal. The genome size of *F.*x *ananassa* and the four wild relatives were estimated, based on a *k*-mer = 17 frequency of the Illumina reads, by using Jellyfish ver. 1.1.6.^31^

### 2.5. Assembly of the *F.* *x* *ananassa* reference genome

The 454 reads were assembled using Newbler 2.7 (Roche Diagnostics) in a heterozygotic mode (Fig. 1). The heterozygous bases between the homoeologous or heterozygous genomes were eliminated by the overlap layout consensus method, with sequence identity of 90%. The gaps on the scaffolds were closed by GapCloser 1.10 (*p* = 31) (http://soap.genomics.org.cn/soapdenovo.html) for the Illumina reads. In parallel, all the *F.*x *ananassa* Illumina reads were assembled using SOAPdenovo v1.05^32^ with *k*-mer = 75. After the assembly of the Illumina reads, the gaps on the scaffolds were closed by GapCloser 1.10 (*p* = 31). Illumina-specific scaffolds and contigs, when compared with the 454 scaffolds, were selected by conducting BLASTN^33^ searches with an *E*-value cut-off of 1*E*−10. To eliminate heterozygous bases, the Illumina-specific scaffolds and contigs were re-assembled according to the approach employed to assemble the fire ant genome.^34^ First, the sequences of Illumina-specific scaffolds and unassembled contigs were divided into 300-bp lengths with 200-bp overlaps. The divided sequences were then re-assembled using Newbler 2.7 in the heterozygotic mode. In cases when the divided sequences were not joined by Newbler 2.7, they were merged based on the overlapping sequences.

Probable contaminating sequences on the 454 scaffolds and the Illumina-specific sequences were identified and removed using BLASTN searches against the chloroplast genome sequence of *F. vesca* (accession number: NC_015206.1), mitochondrial genome sequence of *Arabidopsis thaliana* (accession number: NC_001284.2), bacterial genome sequences registered in NCBI (http://www.ncbi.nlm.nih.gov), and vector sequences from UniVec (http://www.ncbi.nlm.nih.gov/tools/vecscreen/univec/) with an *E*-value cut-off of 1*E*−10 and length coverage of ≥10%. The cleaned Illumina scaffolds, contigs, and singlets were integrated with the 454 scaffolds and designated as FANhybrid_r1.2.

### 2.6. Assembly of Illumina reads from *F. x ananassa* and wild species

The assembly of Illumina reads from *F.*x *ananassa* was performed along with that of reads from the four wild species, *F. iinumae*, *F. nipponica*, *F. nubicola*, and *F. orientalis* (Fig. 2). The Illumina reads in each species were assembled using SOAPdenovo v1.05 with *k*-mer = 75. The gaps on the scaffolds in each species were closed by GapCloser 1.10 (*p* = 31). Probable contaminating sequences were identified and removed as described in the above section. The cleaned scaffolds and contigs ≥300 bp in length were designated FAN_r1.1 (*F.*x *ananassa*), FII_r1.1 (*F. iinumae*), FNI_r1.1 (*F. nipponica*), FNU_r1.1 (*F. nubicola*), and FOR_r1.1 (*F. orientalis*).

### 2.7. Repetitive sequences

Probable repetitive sequences in the assembled genome of the *Fragaria* species were identified by RepeatScout^35^ with default parameters based on the discovery of repetitive substrings in the sequences. In parallel, similarity searches and repeat masking were performed by RepeatMasker (http://www.repeatmasker.org) on the assembled genome sequences of the *Fragaria* species against known repetitive sequences registered in RepBase.^36^ To identify novel repetitive sequences, the assembled genome sequences, with known repetitive sequences masked, were subjected to second-round similarity search by RepeatMasker against probable repetitive sequences detected by RepeatScout. The SSR motifs with equal or greater numbers of defined repeats were searched for in exons, introns, and entire regions on the assembled genomes using SciRoKo with misa mode.^37^ The defined numbers of repeats in mono-, di-, tri-, tetra-, penta-, and hexa-motifs were 12, 6, 7, 5, 5, and 5, respectively.

### 2.8. Assignment of RNA-encoding genes

Transfer RNA genes were predicted by using tRNAscan-SE ver. 1.23^38^ with default parameters. Ribosomal RNA genes were identified by BLAT searches in BLAST output format, with an *E*-value cut-off of 1*E*−10 and length cut-off of 70 bp, using 5.8S rRNA (accession number: X15589.1), 18S rRNA (accession number: X15590.1), and 26S rRNA (accession number: X58118.1), in *F.*x *ananassa*.

### 2.9. Gene prediction and annotation

The FANhybrid_r1.2 and the five Illumina-assembled genomes were subjected to gene prediction and modelling using Augustus 2.7^39^ with a training set from *A. thaliana* and the following parameters; gene model = partial; protein, introns, start, stop, cds, coding seq, gff3, and UTR = on, and alternatives-from-evidence and alternatives-from-sampling = true. The genes related to transposable elements (TEs) were predicted according to the domain and product names of genes identified by similarity search in two databases, the Interpro database^40^ and the NCBI's non-redundant protein sequence (NR) database (http://www.ncbi.nlm.nih.gov). Similarity searches in the former and later databases were performed by InterProScan^41^ and BLASTX^33^ with an *E*-value cut-off of 1.0 and 1*E*−10, respectively. The Gypsy-type TEs were further identified by a search using the hmmscan module in HMMER 3.0^42^ against the hidden Markov model in the Gypsy Database 2.0.^43^

### 2.10. Comparative analysis between *F. x ananassa* and wild species

BLAT searches^44^ were performed for the sequences in FANhyblid_r1.2 against the pseudomolecules of *F. vesca* (v1.1) with −minscore = 100 and −minIdentity = 95. Subsequently, the sequences in FANhybrid_r1.2 were aligned with the genomic sequences of *F. vesca* based on the results of the BLAT searches. Furthermore, the hit regions were selected according to the following criteria: (match score + number of gaps in query) × 100/query length (%) ≥ 80, hit region in query × 100/query length (%) ≥ 80, match score/query length ≥ 25, 80 ≤ (hit region in subject × 100/query length) ≤ 120. The best alignments in each query sequence were identified approximately roughly identified using the pslReps program^44^ in BLAT with –minCover = 0.20. Validation and modification of the results were manually performed by eye. Furthermore, similarity searches were performed for the five Illumina-assembled genome sequences against FANhybrid_r1.2 by BLAT, and these sequences were aligned with the genome sequences of FANhybrid_r1.2 using the pslReps program as described above. In parallel, the BLASTN searches with an *E*-value cut-off of 1*E*−10 were performed between each possible pair of assembled genomes to investigate sequence similarity across the assembled genome sequences.

BLAT searches were also performed for the five assembled genomes against the 34 809 candidate genes estimated on the *F. vesca* genome^15^ with the same parameters as described above. In addition, a total of 104 annotated candidate genes in *F. vesca*^13^ were selected for further investigation of degree of duplication in the assembled *Fragaria* genome sequences. Similarity searches between these 104 genes and the assembled sequences were performed by the FASTA36 program,^45^ based on a cut-off value of 90% identity. In parallel, Illumina-assembled genes predicted by Augustus 2.7 were subjected to cluster analysis with the 34 809 *F. vesca* candidate genes by using CD-hit v4.6.1^46^ with the following parameters: *c* = 0.8, aS = 0.1.

## 3. Results

### 3.1. Phylogenetic analysis of *Fragaria* species with SSR markers

A total of 632 SSR markers, which generated 954 loci mapped onto the consensus map of *F.*x *ananassa*,^9^ were employed for phylogenetic analysis of 20 wild *Fragaria* species and 4 *F.*x *ananassa* varieties. The average number of observed allelic peaks per marker was 11.1, ranging from 1 to 57 in all the tested species, with an average of 1.8 in each line, ranging from 1.0 in *Fragaria daltoniana* to 3.2 in *F.*x *ananassa* ‘Hokowase’. The genetic distances between all combinations of any two lines were calculated based on the presence or absence of 10 830 allelic peaks derived from the 632 markers. The genetic distances among the 24 lines ranged from 0.07 to 0.33 (Supplementary Table S4). On the constructed phylogenetic tree, the 24 lines were classified into four clusters (Supplementary Fig. S1A). The octoploids were clearly distinguished from *Fragaria* species of other ploidy (cluster C). Among the octoploids, two wild species, *F. virginiana* and *F. chiloensis*, were distinguishable from the cultivated species, *F.*x *ananassa*. The clusters A, B, and D consisted of six *Fragaria* species each. The relative genetic relations did not show large differences between a phylogenetic trees constructed with all samples and di-, tetra-, or octoploid-specific trees. Based on the phylogenetic analysis, the following four wild species were selected as representatives of Clusters A, B, and D for subsequent whole-genome sequencing: *F. nipponica* (Cluster A), *F. iinumae* (Cluster B), *F. nubicola* (Cluster D), and *F. orientalis* (Cluster D). Plural species were selected from Cluster D, because the cluster showed the closest genetic distance to Cluster C.

Genetic distances were investigated for each of the LGs on the consensus map according to the locations of the tested markers^9^ (Supplementary Table S5). The mean genetic distances between *F.*x *ananassa* ‘Reikou’ and the 20 wild relatives ranged from 0.24 (LG3A) to 0.34 (LG3D). In comparison, among the homoeologous groups (HGs), HG5 showed the least variation between the corresponding LGs (0.28–0.30), whereas HG3 showed the greatest variation between the corresponding LGs (0.24–0.34). *Fragaria vesca* and *F. chinensis* showed closer genetic distances to *F.*x *ananassa* than any other diploid wild species for every LG except LG5D, for which the closest diploid species was *F. iinumae*.

### 3.2. Genome sequencing and quality control

A total of 6 211 718 reads were obtained from *F.*x *ananassa* SE and PE libraries using the Roche 454 GS FLX+ platform (Supplementary Table S3). The number of obtained Illumina reads in *F.*x *ananassa*, *F. iinumae*, *F. nipponica*, *F. nubicola*, and *F. orientalis* were 4 219 377 380, 1 078 824 432, 1 075 841 020, 1 074 353 610, and 1 050 815 546, respectively. After quality control, 33% of the Illumina reads were excluded from further analysis. Consequently, the total bases that were subjected to subsequent analysis were 276.6, 70.8, 72.2, 74.3, and 74.5 Gb, in *F.*x *ananassa*, *F. iinumae*, *F. nipponica*, *F. nubicola*, and *F. orientalis*, respectively.

### 3.3. Estimation of genome size based on the Illumina reads

The genome sizes of *F.*x *ananassa* and the four wild relatives were estimated based on a *k*-mer = 17 frequency of the Illumina reads (Supplementary Fig. S2). The genome sizes of the five *Fragaria* species were estimated as follows based on the distributions of distinct *k*mers along with consideration of the *F. vesca* genome size (209.8 Mb)^13^ and polyploidy in each species: *F.*x *ananassa* = 692 Mb, *F. iinumae* = 221 Mb, *F. nipponica* = 208 Mb, *F. nubicola* = 202 Mb, and *F. orientalis* *=* 349.3 Mb.

### 3.4. Assembly of the *F. x ananassa* reference genome

All 454 reads were assembled using Newbler 2.7 in the heterozygotic mode (Fig. 1). During assembly, heterozygous bases on the 454 reads were adopted based on the comparison of depth and accuracy between the bases. After the gaps were closed by GapCloser 1.0, a total of 7598 scaffolds consisting of 101 218 723 bp with 19 520 552 N were obtained (Supplementary Table S6). The longest contig was 348 406 bp, and N50 was 46 803 bp.

In parallel, a total of 4 602 723 scaffolds and contigs consisting of 1 301 006 845 bp were generated using SOAPdenovo v1.05 and GapCloser 1.10. After the BLASTN search of the Illumina sequences against the 454 scaffolds, 1 153 521 Illumina-specific contigs were obtained, comprising 213.5 Mb. To assemble the Illumina-specific sequences using Newbler 2.7, the sequences that were longer than 300 bp were divided into 300-bp long sequences with 200-bp overlaps. As a result, a total of 1 437 769 Illumina sequences were re-assembled onto 40 416 215 bp (Supplementary Table S6). In addition, 137 608 unassembled singlets were obtained, including 10 330 repeats and 44 686 outliers. The total length of Illumina-specific sequences was 72 010 849 bp, and the N50 was 411 bp. The integrated 454 scaffolds and Illumina-specific sequences were qualified as a reference genome for *F.*x *ananassa*, designated FANhybrid_r1.2. The number of sequences, total length, N50, and GC% of the FANhybrid_r1.2 were: 211 588 sequences, 173 229 572 bp, 5137 bp, and 38.4%, respectively (Table 1).

**Table 1.** Statistics regarding the assembled genome sequences for *F.*x *ananassa* and four wild *Fragaria* species

	FANhybrid_r1.2	FAN_r1.1	FII_r1.1	FNI_r1.1	FNU_r1.1	FOR_r1.1
Number of sequences	211 588	625 966	117 822	215 024	210 780	323 163
Total length (bp)	173 229 572	697 765 214	199 627 645	206 414 979	203 686 576	214 184 046
Average length (bp)	819	1115	1694	960	966	663
Maximum length (bp)	348 406	51 398	36 387	32 113	27 253	12 564
N50 length (bp)	5137	2201	3309	1275	1291	722
A	47 398 175	201 607 452	60 846 197	63 581 246	62 914 664	66 367 041
T	47 314 079	201 232 679	60 764 847	63 483 905	62 877 129	66 179 649
G	29 454 229	127 672 886	38 950 399	39 626 127	38 930 937	40 738 935
C	29 542 534	127 932 068	39 045 461	39 709 257	38 949 864	40 883 595
N	19 520 555	39 320 129	20 741	14 444	13 982	14 826
Total (A + T + C + G)	153 709 017	658 445 085	199 606 904	206 400 535	203 672 594	214 169 220
GC% (GC/ATGC)	38.4	38.8	39.1	38.4	38.2	38.1

### 3.5. Illumina genome assembly in *F. x ananassa* and wild *Fragaria* species

A total of 276.6 Gb of *F.*x *ananassa* Illumina reads were assembled by SOAPdenovo v1.05 and GapCloser 1.10. After excluding sequences of contaminating DNA, the number of sequences and total length of the Illumina-assembled sequences were 4 366 193 sequences and 1 264 283 529 bp, respectively (Supplementary Table S7). The assembled sequences that were shorter than 300 bp in length accounted for 86% of the total were excluded from further analysis. The selected sequences were totalled 697 765 214 bp with 39 320 129 Ns, and designated as FAN_r1.1. N50 and maximum length of the FAN_r1.1 were 2201 and 51 398 bp, respectively (Table 1).

In parallel, *de novo* genome sequence assembly was performed for Illumina reads of the four wild species using SOAPdenovo v1.05 and GapCloser 1.10. As with *F.*x *ananassa*, assembled sequences of <300 bp in length were excluded from further analysis. The remaining sequences of *F. iinumae*, *F. nipponica*, *F. nubicola*, and *F. orientalis* were designated as FII_r1.1, FNI_r1.1, FNU_r1.1, and FOR_r1.1, respectively (Table 1 and Supplementary Table S7). The total length and N50 in the assembled genome sequences of the four wild species ranged from 199.6 (FII_r1.1) to 214.2 Mb (FOR_r1.1), and 722 (FOR_r1.1) to 3309 bp (FII_r1.1), respectively. GC% ranged from 38.1% (FOR_1.1) to 39.1% (FII_r1.1).

### 3.6. Repetitive sequences

The total length of repetitive sequences identified by RepeatMasker was 8 697 730 bp in FANhydrid_r1.2 (5.0% of the total length); 328 305 437 bp in FAN_r1.1 (47.1%); 63 285 682 bp in FII_r1.1 (31.7%); 52 580 277 bp in FNI_r1.1 (25.5%); 49 943 156 bp in FNU_r1.1 (24.5%); and 56 272 506 bp in FOR_r1.1 (26.3%) (Supplementary Table S8). When the same approach was applied to the *F. vesca* genome (v1.1), 51 223 702 bp (24.8%) was comprised of repeat sequences. Most of the identified repeats in the assembled genomes, except FANhybrid_r1.2, were novel repeats that were not registered in RepBase. The ratios of the novel repeats to the total lengths of Illumina-assembled genome sequences ranged from 22.6% (FNU_r1.1) to 44.9% (FAN_r1.1), whereas that in the FANhybrid_r1.2 was 3.9%. The ratio of the total length of known interspersed repeats to the total genome length ranged from 0.35% (FANhybrid_r1.2) to 0.94% (FAN_r1.1). The Class 1 long terminal repeat retrotransposons, including *Copia* and *Gypsy* types, were the most frequency observed in the known interspersed repeats.

The total numbers of identified di- to hexa-nucleotide SSRs were 22 456 (FANhybrid_r1.2), 110 251 (FAN_r1.1), 32 487 (FII_r1.1), 35 188 (FNI_r1.1), 34 556 (FNU_r1.1), and 32 340 (FOR_r1.1) (Supplementary Table S9). In each genome, di-nucleotide motifs were the most frequently observed type of SSR, ranging from 57.6% (FANhybrid_r1.2) to 69.6% (FII_r1.1). The (AT)*_n_* motif was the most abundantly observed in the Illumina-assembled genomes, whereas (AG)*_n_* was the most frequent motif in FANhybrid_r1.2.

### 3.7. RNA-encoding genes

The total number of putative tRNA-encoding genes in FANhybrid_r1.2 was 300, which was less than in the *F. vesca* scaffolds (Supplementary Table S10). Large numbers of putative tRNA-encoding genes (1720) were identified in FAN_r1.1, while 424–514 tRNA-encoding genes were predicted in the four wild Illumina-assembled genomes. The ratios of putative tRNA genes encoding amino acids ranged from 83.7% (FOR_r1.1) to 92.4% (FII_r1.1). The total number of predicted rRNA-encoding genes in FANhybrid_r1.2, FAN_r1.1, FII_r1.1, FNI_r1.1, FNU_r1.1, and FOR_r1.1 were 18, 86, 37, 53, 19, and 45, respectively (Supplementary Table S11).

### 3.8. Gene prediction and annotation

The total number of predicted genes in FANhybrid_r1.2 was 45 377; more genes than in *F. vesca* (v1.0) but less than in other assembled genome sequences (Supplementary Table S12). The total sequence length and N50 were 32 959 863 and 1290 bp, respectively. Total numbers of predicted genes in the wild *Fragaria* sequences ranged from 76 760 (FII_r1.1) to 99 674 (FOR_r1.1). Approximately 3.7-fold more total sequence length were identified in FAN_r1.1 compared with FANhybrid_r1.2. The N50 of the five Illumina-assembled genomes ranged from 484 (FOR_r1.1) to 948 bp (FII_r1.1).

The predicted genes were classified into TE genes versus non-TE genes (designated as intrinsic genes, Supplementary Table S13). The following three gene categories were subsequently notated: partial genes (without start or stop codons), pseudogenes (with in-frame stop codons), and short genes (encoding fewer than 49 aa). No notations were given for predicted full-length gene sequences framed by start and stop codons. In FANhybrid_r1.2, 41 730 sequences (92% of the total sequences) were predicted to be intrinsic genes, while the other 3647 (8%) were annotated as TEs. Among the intrinsic genes in FANhybrid_r1.2, 41% did not fall neatly into any of the three categories. This ratio was higher than that in the Illumina-assembled genome sequences.

### 3.9. Comparative analysis between the genomes of cultivated and wild *Fragaria* species

Of the 211 588 sequences in FANhybrid_r1.2, 120 703 showed significant similarity to pseudomolecules of *F. vesca* (v1.1) by BLAT search (Supplementary Table S14). The lengths of the mapped FANhybrid_r1.2 sequences in each of the seven chromosomes ranged from 13 922 608 (Chr1) to 26 401 015 bp (Chr6). The FANhybrid_r1.2 sequences showing significant similarity according to the BLAT search were well aligned across the *F. vesca* pseudomolecules (Supplementary Fig. S3). The mapped FANhybrid_r1.2 sequences included 145 387 993 bp of *F. vesca* pseudomolecules, which covered 70.3% of the total length (Supplementary Table S14). Furthermore, the MEGABLAST^47^ search (identity% ≥90 and *E*-value cut-off of 1*E*−50) was performed for the 90 885 FANhybrid_r1.2 sequences that did not align with the *F. vesca* pseudomolecules, against the wild *Fragaria* genome sequences. Of the 90 885 FANhybrid_r1.2 sequences, 22 145 showed significant similarity against the *F. vesca* genome sequences, whereas 68 740 did not (Supplementary Table S15). A total of 39 692 FANhybrid_r1.2 sequences (total length: 10 742 467 bp) showed no significant similarity to any of the genome sequences, and were concluded to be *F.* x *ananassa-*specific. In parallel, BLASTN searches were performed between all pairwise combinations of the assembled genomes (Supplementary Table S16). The total length of non-homologous sequences in FANhybrid_r1.2 compared with FAN_r1.1 was 1.4 Mb, whereas that in FAN_r1.1 compared with FANhybrid_r1.2 was 281.7 kb. Comparison with each of the five wild species revealed that the ratios of non-homologous sequences to total sequences of FANhybrid_r1.2 and FAN_r1.1 ranged from 4.7% (FNU_r1.1) to 5.6% (FOR_r1.1), and from 1.3% (FNU_r1.1) to 1.8% *(F. vesca* v1.1), respectively. In comparisons between the wild species, the smallest ratio of non-homologous sequences to the total was observed in FNU_r1.1 against FNI_r1.1 (0.66%), whereas the largest ratio was observed in FNI_r1.1 against FII_r1.1 (2.61%).

**Figure 3.**
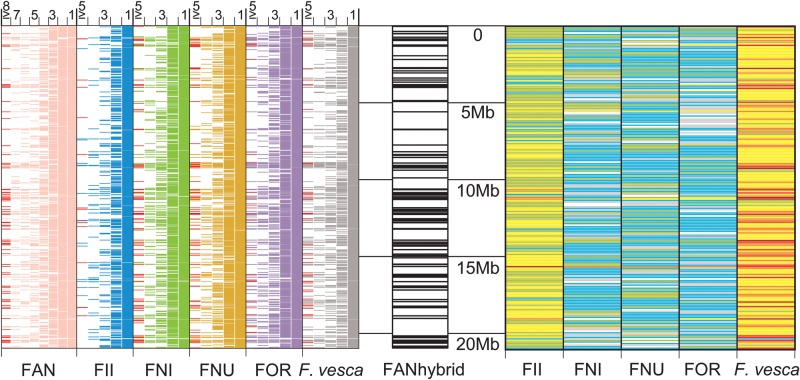
Position and coverage of the Illumina-assembled genome sequences mapped onto the FANhybrid_r1.2 sequence (left), and frequency of the top-hit sequences from the five wild *Fragaria* species against FANhybrid_r1.2 (right). The central black and white bars indicate the genome sequences of FANhybrid_r1.2 aligned based on the homologous sequence positions on *F. vesca* (v1.1) Chr1. Details are described in Supplementary Figs S3 and S4.

A BLAT search mapped 142 699 FAN_r1.1 sequences (61.8% of the total) onto the FANhybrid_r1.2 sequence (Supplementary Table S17). Multiple FAN_r1.1 sequences were often mapped onto the same FANhybrid_r1.2 sequences. The numbers of single, double, triple, and quadruple FAN_r1.1 sequences that mapped onto the same FANhybrid_r1.2 sequences were 64 828 (45.4% of the mapped total), 31 922 (22.4%), 13 656 (9.6%), and 6289 (4.4%), respectively. The FANhybrid_r1.2 sequences that mapped onto five or more FAN_r1.1 sequences tended to be classified as repetitive sequences (Fig. 3 and Supplementary Fig. S4). The sequence of each wild species was then mapped onto the entire genomic sequence of FANhybrid_r1.2. The ratios of single and double sequences from the wild Illumina-assembled genomes that mapped onto the same FANhybrid_r1.2 sequences ranged from 62.4% (FOR_r1.1) to 87.3% (FII_r1.1), and from 9.5% (FII_r1.1) to 18.5% (FOR_r1.1), respectively. The ratios of single and double sequences from the *F. vesca* genome that mapped onto the same FANhybrid_r1.2 sequences were 72.7 and 4.8%, respectively. *Fragaria vesca* generated the largest number of top-hit sequences in each chromosome, followed by *F. iinumae* (Fig. 3 and Supplementary Fig. S5). The BLASTN search was furthermore performed for the FAN_r1.1 against the wild *Fragaria* genome sequences. Of the 625 966 FAN_r1.1 sequences, 5447 (0.9%) were not identified significant similarity to the wild *Fragaria* sequences (Supplementary Table S18). The ratios of top-hit sequences in FII_r1.1, FNI_r1.1, FNU_r1.1, FOR_r1.1, and *F. vesca* (v1.1) to the FAN_r1.1 sequences were 39.1, 8.9, 8.1, 8.2, and 34.8%, respectively.

### 3.10. Comparative analysis between the genes in *F. vesca* and the assembled sequences in the *Fragaria* species

To investigate the degree of gene duplication in each assembled genome, BLAT searches were performed for the six assembled genome sequences against the 34 809 candidate genes identified on the *F. vesca* genome.^15^ The numbers of BLAT hit sequences in FANhybrid_r1.2, FAN_r1.1, FII_r1.1, FNI_r1.1, FNU_r1.1, and FOR_r1.1 were 27 718 (79.6% of the 34 809 candidate genes), 32 120 (92.3%), 27 560 (79.2%), 29 323 (84.2%), 31 869 (91.6%), and 31 901 (91.6%), respectively. Single hits were the most frequently observed in all the assembled genome sequences except FAN_r1.1 (Supplementary Fig. S6a). The distribution of FAN_r1.1 differed from those of the other assembled genome sequences; the peak in frequency was quite broad, and triple hits were the most frequently observed. Of the four wild species, the *F. orientalis* (FOR_r1.1) genome showed broader peaks than the others, whereas sharper peaks were observed in the *F. iinumae* genome (FII_r1.1). Cluster analysis was performed on the gene sequences predicted as ‘intrinsic gene’ and ‘intrinsic gene/partial’ (Supplementary Table S13) in the Illumina-assembled genome sequences of *F.*x *ananassa* and the diploids (Supplementary Fig. S7) against 34 809 *F. vesca* candidate genes. The numbers of gene sequences that did not cluster in any other species were 24 596 (14.2% of the total sequences) in FAN_r1.1; 3989 (6.7%) in FII_r1.1; 6351 (8.8%) in FNI_r1.1; 3399 (4.8%) in FNU_r1.1; and 1492 (4.6%) in *F. vesca*. The BLAT search results were further investigated for 104 genes that were annotated as agriculturally important traits in a previous study^15^ (Supplementary Table S19). The numbers of genes that had no hits to any of the assembled genomes were as follows: 17 (FANhybrid_r1.2), 10 (FII_r1.1), 3 (FNI_r1.1), 7 (FNU_r1.1), and 6 (FOR_r1.1). All the *F. vesca* genes showed hits to the FAN_r1.1 sequences. The distributions of the hit sequences were similar to the BLAT search results against the 34 809 genes, except the highest numbers of hit sequences in FNI_r1.1, and FOR_r1.1 were 2 (Supplementary Fig. S6b).

## 4. Discussion

The phylogenetic analysis with 632 markers revealed that *F. vesca* was the most closely related diploid species to *F.*x *ananassa.* The constructed phylogenetic tree suggested that *F. vesca* and *F. nubicola* were genetically closer to *F.*x *ananassa* than *F. iinumae* and *F. nipponica.* A comparison of the genetic distances between all pairs of the three diploids, *F. iinumae*, *F. nipponica*, and *F. nubicola*, indicated that *F. iinumae* and *F. nipponica* were more closely related than the other pairs. This result diverged from that of a previous report,^10,14^ which distinguished the genome of *F. iinumae* from those of *F. nipponica* and *F. nubicola*. It is considered that differences in investigated regions and scales may be responsible for the inconsistency to the previous studies. We concluded that the four wild species, *F. iinumae*, *F. nipponica*, *F. nubicola*, and *F. orientalis*, were good representatives of the genetic diversity in the genus *Fragaria* and were subjected to further genome sequencing analysis.

The genome size of *F.*x *ananassa* was estimated as 692 Mb, based on the multiplicity of distinct *k*-mers in the Illumina reads. Despite the similarity in size to that determined in previous studies (708–720 Mb),^2,3^ it was less than 4-fold the size of the *F. vesca* genome (209.8 Mb).^13^ Similarly, the genome size of *F. orientalis* (349.3 Mb) was less than 2-fold the size of the *F. vesca* genome. Therefore, we suspected that the genome sizes of *F.*x *ananassa* and *F. orientalis* were underestimated. The estimated genome size of the three diploids, *F. iinumae*, *F. nipponica*, and *F. nubiclola*, was similar to that of *F. vesca*, and we therefore concluded that the estimated values were close to the true values. Most of the species, except *F. orientalis*, showed two peaks in the distribution of the number of distinct *k*-mers, and the distinct peaks detected on larger multiplicity were employed for the genome size estimation. Because not all the sequenced materials were from homozygous lines, the peaks identified on smaller multiplicity were assumed to stem from heterozygous sequences. The peaks employed for genome size estimations in *F.*x *ananassa* and *F. orientalis* were quite broad. The broad shape of the peaks may have resulted from the homoeologous nature of the sequences, and this could also have led to the underestimation of the genome sizes.

The largest obstacle to genome sequence assembly of octoploid *F.*x *ananassa* was the homoeology of the subgenomes. In addition, outcrossing behaviour of *F.*x *ananassa* generates allelic heterozygosity within pairs of homoeologous genomes. Since up to eight heterozygous sequences exist in the *F.*x *ananassa* genome, it was predicted that this heterozygosity would create difficulty in assembling chromosome-specific sequences. Therefore, we tried to construct a virtual reference genome that could integrate genome sequences of homoeologous or heterozygous chromosomes by eliminating heterozygous bases. A number of assembling programmes were developed for the assembly of sequences generated by the NGS platforms. The base strategies were mainly classified into two methods: the overlap graph method and the de Bruijn graph method.^48,49^ The overlap graph method lays out a consensus paradigm based on overlapping sequences, whereas the de Bruijn graph method segments sequence reads into *k*-mers, and then assembles *k*-mers based on paths represented on the graphed reads. Because the overlap graph method assumes similarities between the reads in sequence assembly, it is able to eliminate heterozygous bases. The Newbler 2.7 and SOAPdenovo v1.05 are typical assemblers employing the overlap graph method and the de Bruijn graph method, respectively. Therefore, Newbler 2.7 was used for the assembly of the reference genome sequences, whereas SOAPdenovo v1.05 was used for the assembly of the sequence that maintained heterozygosity. The total length of the assembled genome, FANhybrid_r1.2, was 173.2 Mb, which was shorter than that of *F. vesca*. The N50 of the 454 scaffolds was 46 803 bp, whereas that of the Illumina scaffolds and contigs was 411 bp. Therefore, FANhybrid_r1.2 consisted of long 454 scaffolds and short Illumina sequences.

The total length of all Illumina-assembled genome sequences in *F.*x *ananassa* was 1264.3 Mb, which was ∼2-fold the genome size estimated by the Jellyfish program, and more than 6-fold the genome size of *F. vesca*. The N50 was quite short, 406 bp. The long total length and short N50 suggested that assembly of the Illumina reads was obstructed by large numbers of heterozygous sequences. Hence, contigs shorter than 300 bp were excluded from further analysis. As a result, the total length of FAN_r1.1 was reduced to 697.8 Mb, which was close to the length estimated by Jellyfish. The result of the similarity search against the candidate genes in *F. vesca* (Supplementary Fig. S6) indicated that high heterozygosity was maintained in FAN_r1.1 sequences. As with FAN_r1.1, the total lengths of Illumina-assembled genome sequences in the three diploids were similar to the genome size estimated by Jellyfish. However, the total length of *F. orientalis* (FOR_r1.1) was 214.2 Mb, which was ∼61% of the genome size as estimated by Jellyfish. This result indicated a possibility of over-exclusion of sequences caused by the removal of sequences <300 bp in length.

The total lengths of repeat sequences in the four wild Illumina-assembled genome sequences were close to that of *F. vesca*, whereas the FAN_r1.1 sequence was ∼6.4 times that of *F. vesca* (Supplementary Table S8). This result implied that the total length of repeat sequences in FAN_r1.1 was overestimated due to the high heterozygosity in the sequences. In contrast, the total length of the repeat sequences in FANhybrid_r1.2 was quite short, 0.17 that of *F. vesca*. We considered that excessive integration had occurred as a result of the elimination of heterozygous bases through the use of the heterozygotic mode in Newbler 2.7. Similar patterns were observed in the numbers of tRNAs (Supplementary Table S10) and the length and numbers of candidate genes predicted by Augustus 2.7 (Supplementary Table S12). The frequency of SSRs ranged from 12.7 (FANhybrid_r1.2) to 16.8 (FNI_r1.1) per 100 kb across the five assembled genome sequences. This agreement in observed SSR frequency suggested that SSR identification was not affected by the heterozygosity in the assembled genome sequences. An SSR frequency in intron regions was 2- to 3-fold that in exon regions. Liu *et al.*^50^ reported loss of rDNA site numbers in octoploids by fluorescence *in situ* hybridization using 5S and 25S rDNA probes. However, we found no clear evidence of structural changes in the genomes during the evolutionary transition from diploids to octoploids based on the features of the assembled genome sequences.

The BLAST analysis between the whole assembled genome sequences (Supplementary Table S16) showed that sequences in FAN_r1.1 covered larger regions in the *F.* x *ananassa* genome than those in FANhybrid_r1.2. The total length of non-homologous sequences was only 281 769 bp in FAN_r1.1 against FANhybrid_r1.2, whereas it was 1.4 Mb in FANhybrid_r1.2 against FAN_r1.1. The ratio of non-homologous sequence to total sequence did not exceed 3% in comparisons between any two pairs of assembled sequences. It was predicted that the percentages of non-homologous sequence between the four subgenomes in *F.*x *ananassa* (A, A′, B, and B′) would reflect those between the ancestral species. Therefore, we estimated that the percentage of non-homologous sequence between the four subgenomes did not exceed 3%.

Based on the results of the BLAT analysis, 57% of the sequences in FANhybrid_r1.2 aligned with *F. vesca* pseudomolecules, while the other 43% of the sequences did not. The large number of non-aligning sequences in the BLAT analysis may be related to the strict thresholds used (i.e. the total length of the subject sequence must show significant similarity over 80–120% of the length of the query sequence). In the Illumina-assembled genomes, multiple sequences were often mapped onto the same FANhybrid_r1.2 sequences, and this might reflect the heterozygosity of the corresponding regions. This multiple mapping was observed across the genome sequence, and we concluded that the heterozygosity was randomly distributed in *F.* x *ananassa* and the four wild species sequenced in this study. In comparisons of the numbers of top-hit sequences of the five wild species, slight difference was observed between the results of BLAT analysis against FANhybrid_r1.2 and BLASTN search against FAN_r1.1. In the result of BLAT analysis, *F. vesca* showed the highest numbers of the top-hit sequences (Supplementary Fig. S5), while nearly equal ratio was observed in *F. iinumae* (39.1%) and *F. vesca* (34.8%) in the BLASTN search (Supplementary Table S18). We considered that the result of BLASTN search was more accurate, because the result of BLAT analysis was affected by the length of assembled sequences in the subject (wild species) sequences. The result of BLASTN search suggested that the genomes of *F. vesca* and *F. iinumae* equally contributed as progenitors of *F.* x *ananassa*, and agreed with previous studies.^3,10,14^ On the other hand, 14.2% of the FAN_r1.1 intrinsic genes were predicted as unique genes in *F.* x *ananassa*. These results suggested that the genome of *F.* x *ananassa* has diverged from the other *Fragaria* species during the process of evolution.

In this study, we dissected the octoploid *F.* x *ananassa* genome through comparisons between reference and heterozygous genome sequences in *F.* x *ananassa* and sequencing analysis of the genomes of wild *Fragaria* relatives. To our knowledge, this is the first genomic analysis of a polyploid species, and we expect that this approach will be applied to the genomic analysis of other polyploid species. On the other hand, one remarkable issue remained; that is, the distinction between homoeologous and allelic heterozygous sequences. The small percentages of non-homologous sequence across the assembled genomes suggested that most of the genes in *F.* x *ananassa* have homoeologous sequences in the genome. To distinguish subgenome-specific sequences, we considered that introducing segregation analysis into the genome sequence assembly would provide a more effective approach. Therefore, S_1_ progenies of ‘Reikou’ were subjected to further analysis. The results obtained should contribute to progress in the genomic and genetic analyses of the octopolyploid species, *F.* x *ananassa*.

## 5. Database

The genome assembly data, annotations, and gene models of *F.*x *ananassa* and wild *Fragaria* species are available at the Strawberry GARDEN (http://strawberry-garden.kazusa.or.jp). All sequence data (assembled sequences and genome sequence reads by NGSs) are available through the international databases (DDBJ/GenBank/EMBL) under the umbrella project number PRJDB1445. The accession numbers of assembled sequences, FANhybrid_r1.2, FAN_r1.1, FII_r1.1, FNI_r1.1, FNU_r1.1, and FOR_r1.1 are BATS01000001-BATS01220286, BATT01000001-BATT01714282, BATU01000001-BATU01118549, BATV01000001-BATV01215530, BATW01000001-BATW01211274, and BATX01000001-BATX01323675, respectively. The genome sequence reads obtained by Roche 454 GS FLX+ and Illumina GAII/HiSeq are available from the DDBJ Sequence Read Archive (DRA) under the accession number DRA001114.

## Funding

This work was supported by the Kazusa DNA Research Institute Foundation.

## Supplementary Material

Supplementary Data
