# Supplementary material for: Dissection of the Octoploid Strawberry Genome by Deep Sequencing of the Genomes of Fragaria Species
Source: DNA Res. 2013 Nov 26;21(2):169–81. doi: 10.1093/dnares/dst049 (PMC3989489; doi:10.1093/dnares/dst049)
Supplement: Supplementary Data [file supp_21_2_169__index.html]

Dissection of the Octoploid Strawberry Genome by Deep Sequencing of the Genomes of Fragaria Species — Dissection of the Octoploid Strawberry Genome by Deep Sequencing of the Genomes of Fragaria Species — Supplementary Data 

# Dissection of the Octoploid Strawberry Genome by Deep Sequencing of the Genomes of *Fragaria* Species

## Supplementary Data

Supplementary Data

**Files in this Data Supplement:**

- Supplementary Figure 4 - pdf file
- Supplementary Figure 5 - pdf file
- Supplementary Figure 1 - ppt file
- Supplementary Figure 2 - ppt file
- Supplementary Figure 3 - ppt file
- Supplementary Figure 6 - ppt file
- Supplementary Figure 7 - ppt file
- Supplementary Tables - xls file
