# Supplementary material for: Dissection of the Octoploid Strawberry Genome by Deep Sequencing of the Genomes of Fragaria Species
Source: DNA Res. 2013 Nov 26;21(2):169–81. doi: 10.1093/dnares/dst049 (PMC3989489; doi:10.1093/dnares/dst049)
Supplement: Supplementary Data [file supp_dst049_dst049supp_fig4.pdf]

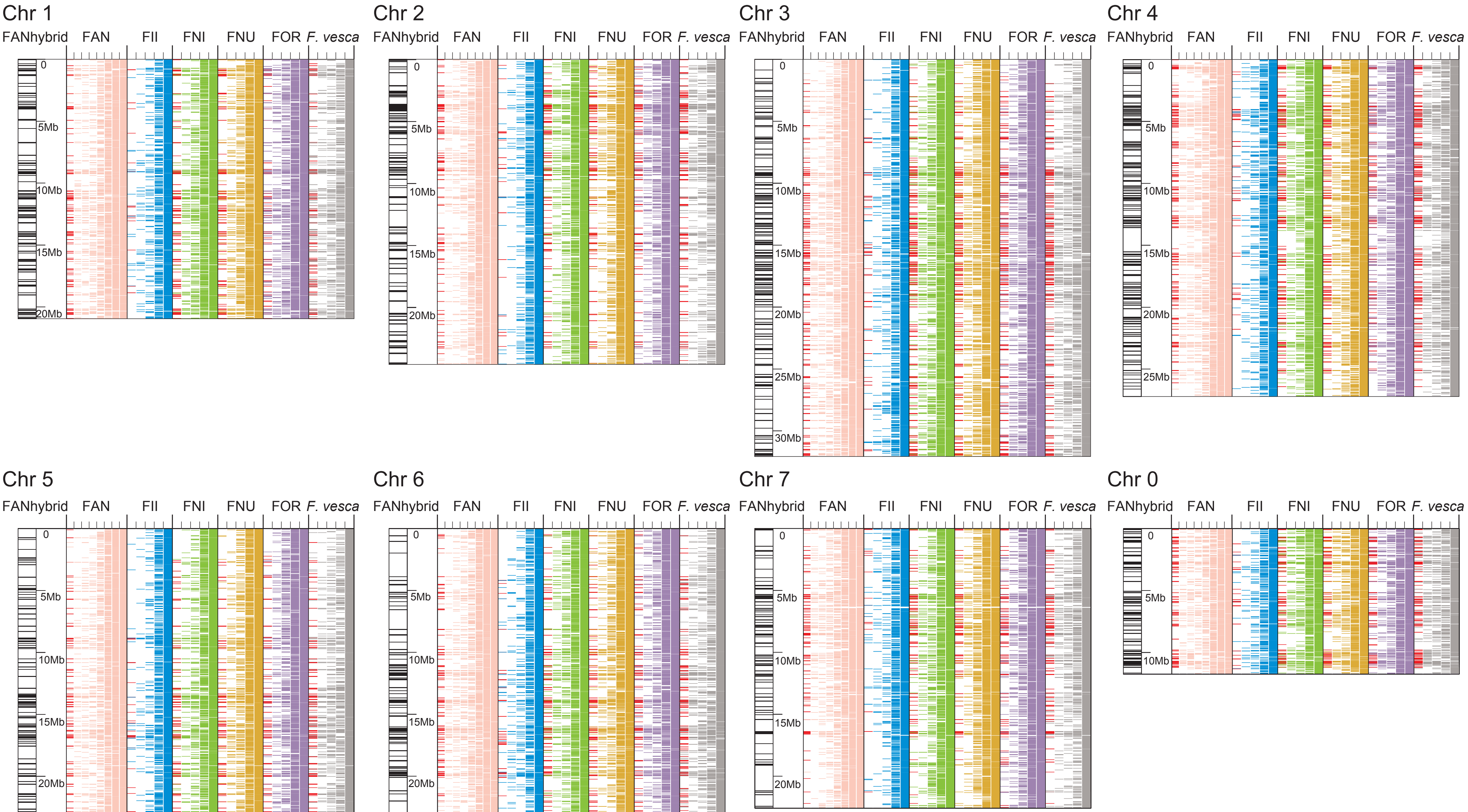

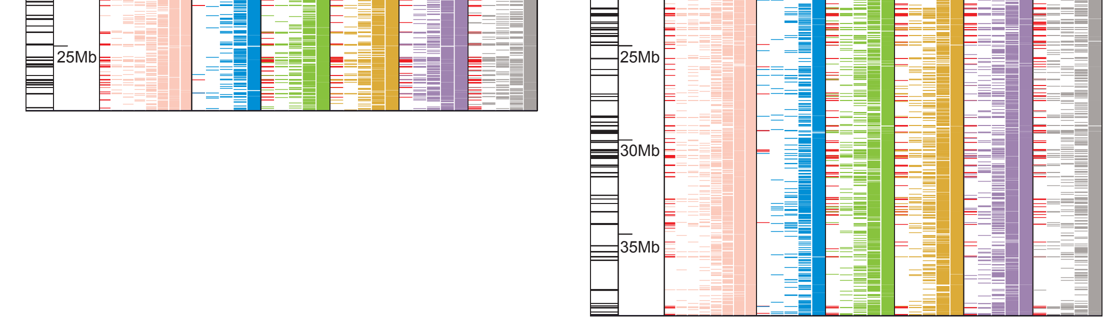

Supplemental Figure S4: Position and coverage of the Illumina-assembled genome sequences mapped onto the FANhybrid_r1.2 sequences. The left-most bars indicate FANhybird_r1.2 genome sequences aligned with the genome of *F. vesca* (v1.1). Black bars represent repeat sequences. Pink, blue, green, yellow, purple, and grey bars indicate absence of homologous sequences between FANhybrid_r1.2 and FAN_r1.1, FII_r1.1, FNI_r1.1, FNU_r1.1, FOR_r1.1, and F. vesca (v1.1), respectively. The X axis on each colored bar shows the mapped coverage of the sequences. In FAN_r1.1, the scales on the X axis from right to left represent one to eight fold coverage. In the other assembled genome sequences, the scales on the X axis from right to left show one- to five-fold coverage. Sequences with absence of homologous sequences with more than eightfold (FAN_r1.1) or five-fold (other assembled genomes) coverage are shown by red bars.
