# Supplementary material for: Dissection of the Octoploid Strawberry Genome by Deep Sequencing of the Genomes of Fragaria Species
Source: DNA Res. 2013 Nov 26;21(2):169–81. doi: 10.1093/dnares/dst049 (PMC3989489; doi:10.1093/dnares/dst049)
Supplement: Supplementary Data [file supp_dst049_dst049supp_fig5.pdf]

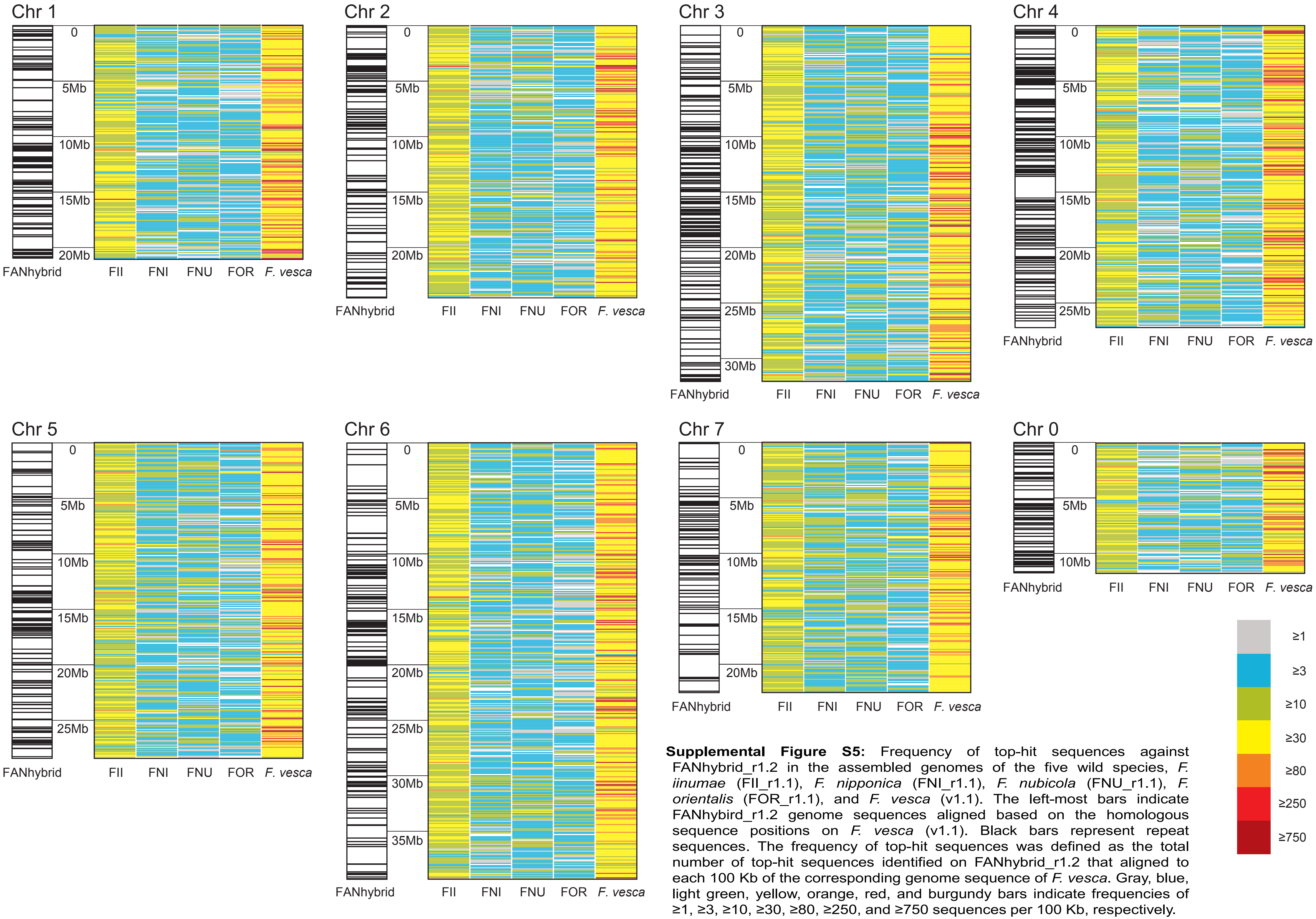

Supplemental Figure S5: Frequency of top-hit sequences against FANhybrid_r1.2 in the assembled genomes of the five wild species, *F. iinumae* (FII_r1.1), *F. nipponica* (FNI_r1.1), *F. nubicola* (FNU_r1.1), *F. orientalis* (FOR_r1.1), and *F. vesca* (v1.1). The left-most bars indicate FANhybird_r1.2 genome sequences aligned based on the homologous sequence positions on *F. vesca* (v1.1). Black bars represent repeat sequences. The frequency of top-hit sequences was defined as the total number of top-hit sequences identified on FANhybrid_r1.2 that aligned to each 100 Kb of the corresponding genome sequence of *F. vesca*. Gray, blue, light green, yellow, orange, red, and burgundy bars indicate frequencies of ≥1, ≥3, ≥10, ≥30, ≥80, ≥250, and ≥750 sequences per 100 Kb, respectively.
