# Supplementary material for: Dissection of the Octoploid Strawberry Genome by Deep Sequencing of the Genomes of Fragaria Species
Source: DNA Res. 2013 Nov 26;21(2):169–81. doi: 10.1093/dnares/dst049 (PMC3989489; doi:10.1093/dnares/dst049)
Supplement: Supplementary Data [file supp_dst049_dst049supp_fig1.ppt]

## Slide 1
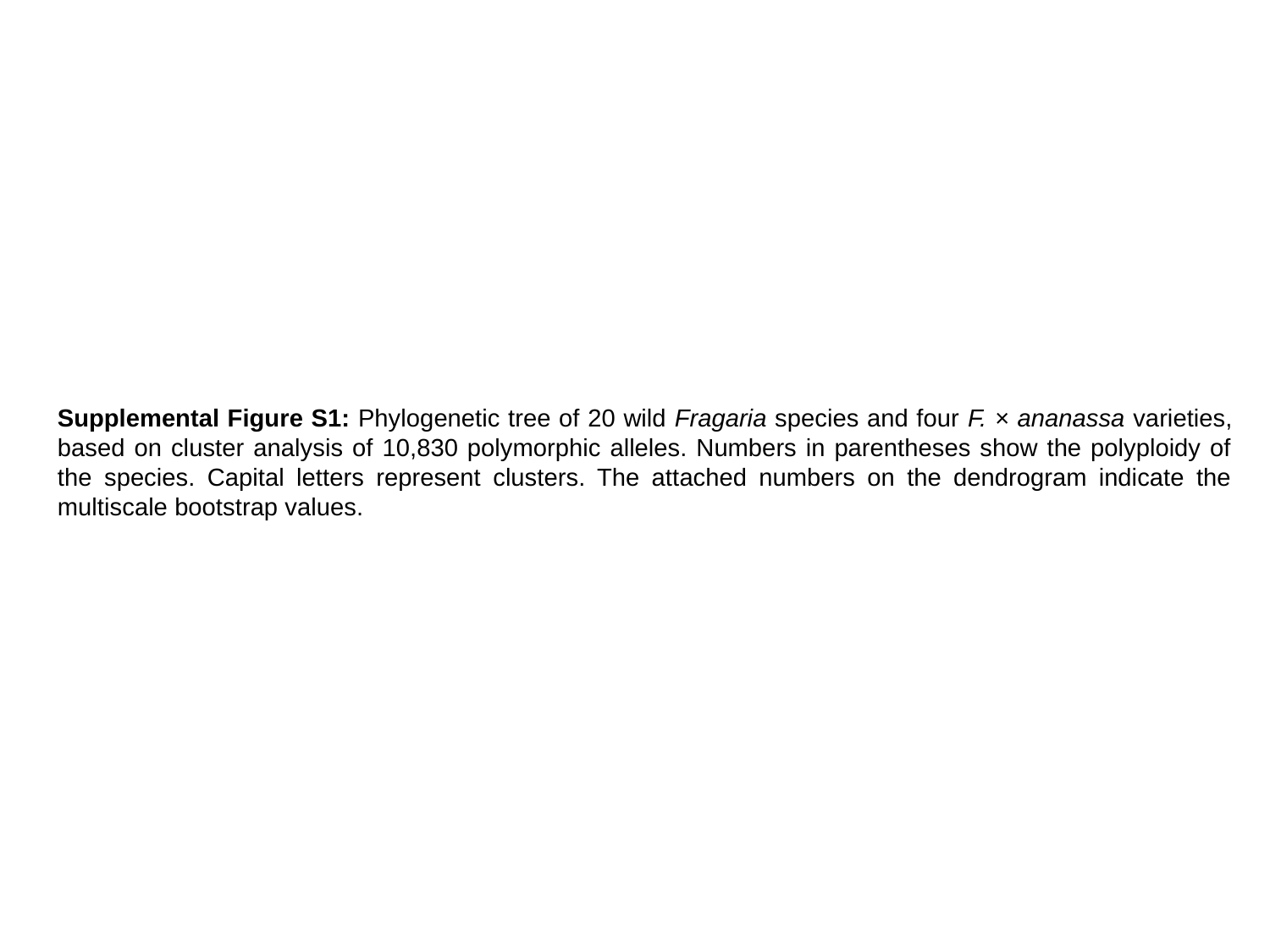

Supplemental Figure S1: Phylogenetic tree of 20 wild Fragaria species and four F. × ananassa varieties, based on cluster analysis of 10,830 polymorphic alleles. Numbers in parentheses show the polyploidy of the species. Capital letters represent clusters. The attached numbers on the dendrogram indicate the multiscale bootstrap values.

## Slide 2
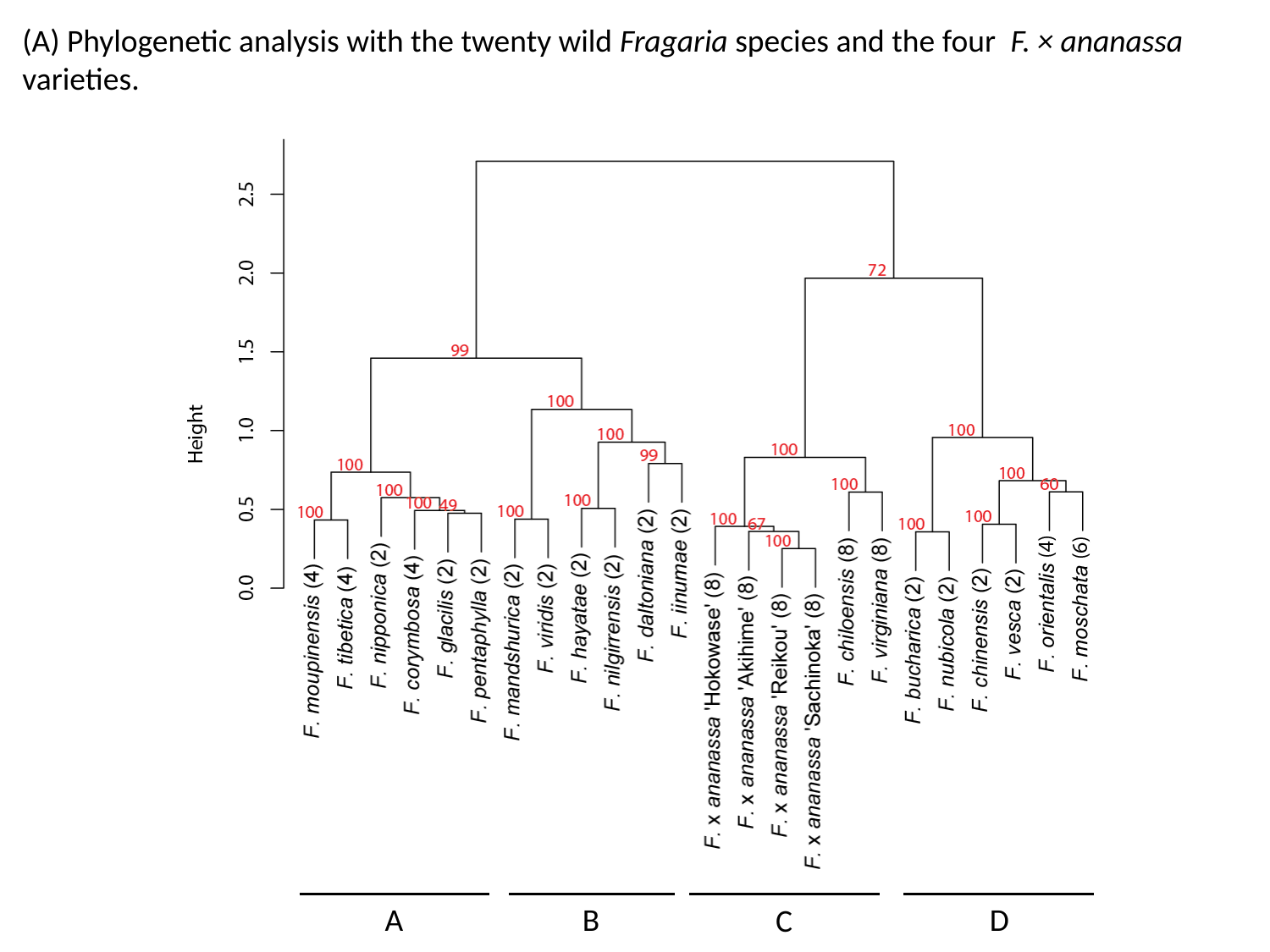

(A) Phylogenetic analysis with the twenty wild Fragaria species and the four F. × ananassa varieties.

## Slide 3
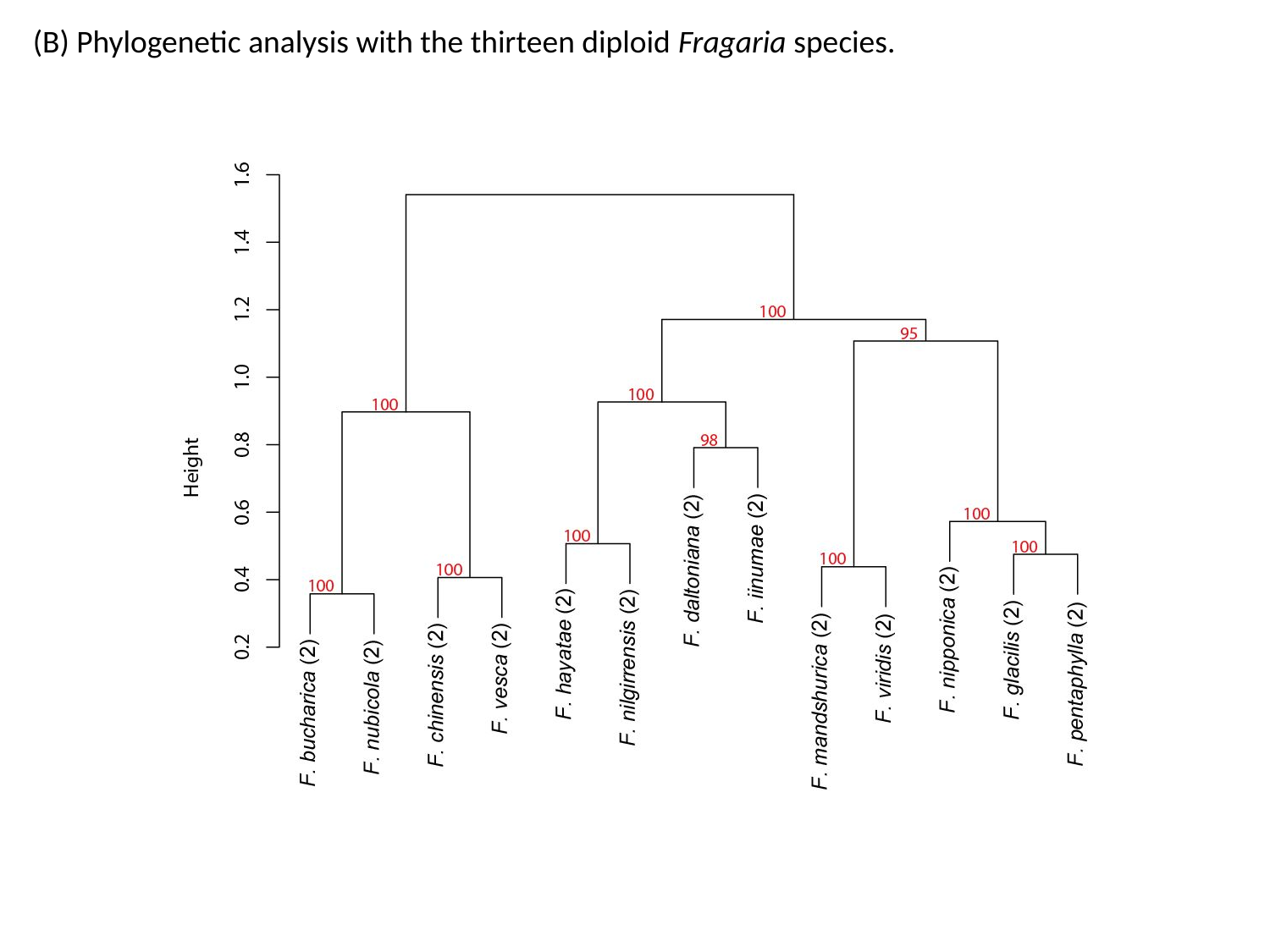

(B) Phylogenetic analysis with the thirteen diploid Fragaria species.

## Slide 4
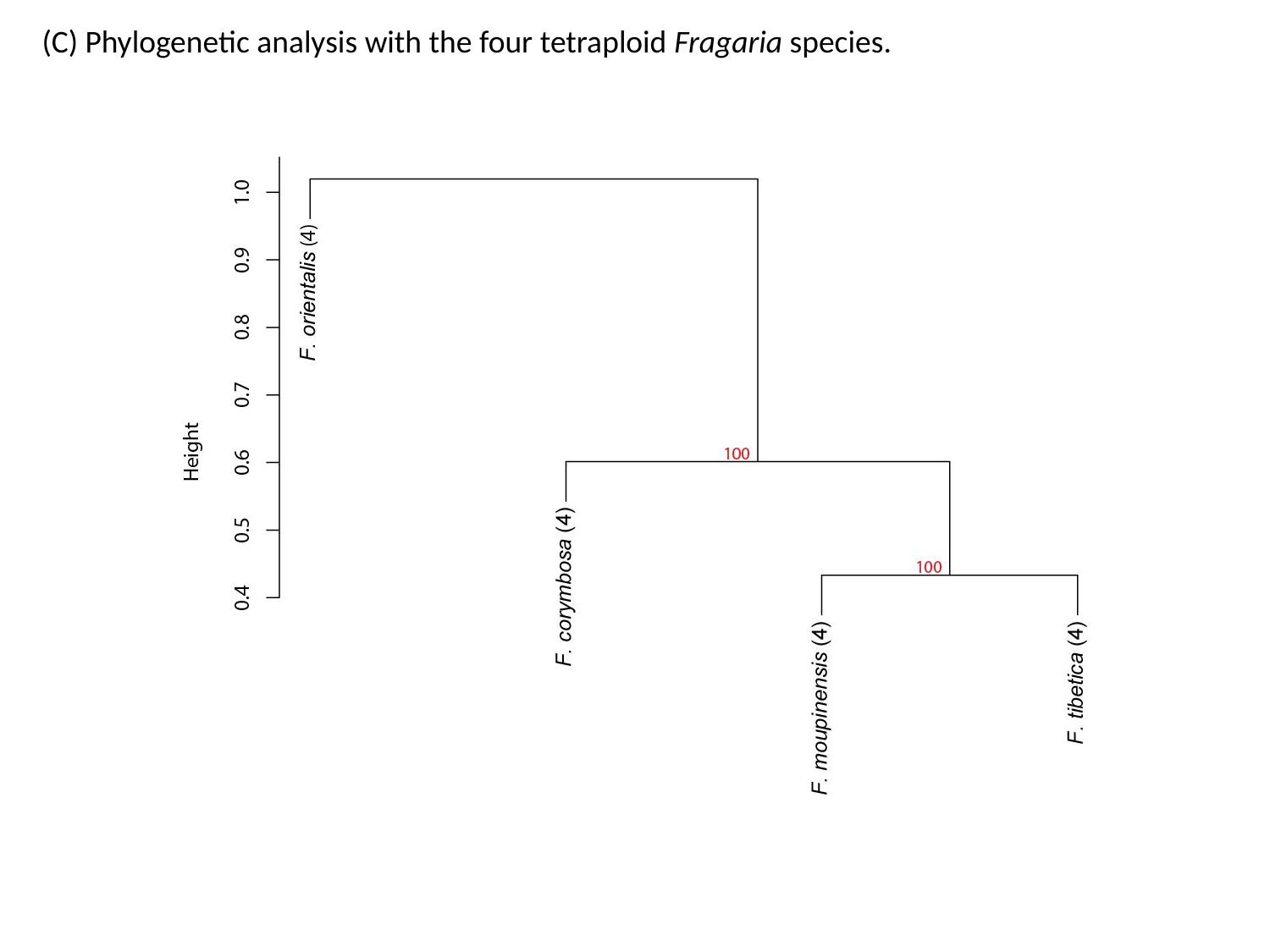

(C) Phylogenetic analysis with the four tetraploid Fragaria species.

## Slide 5
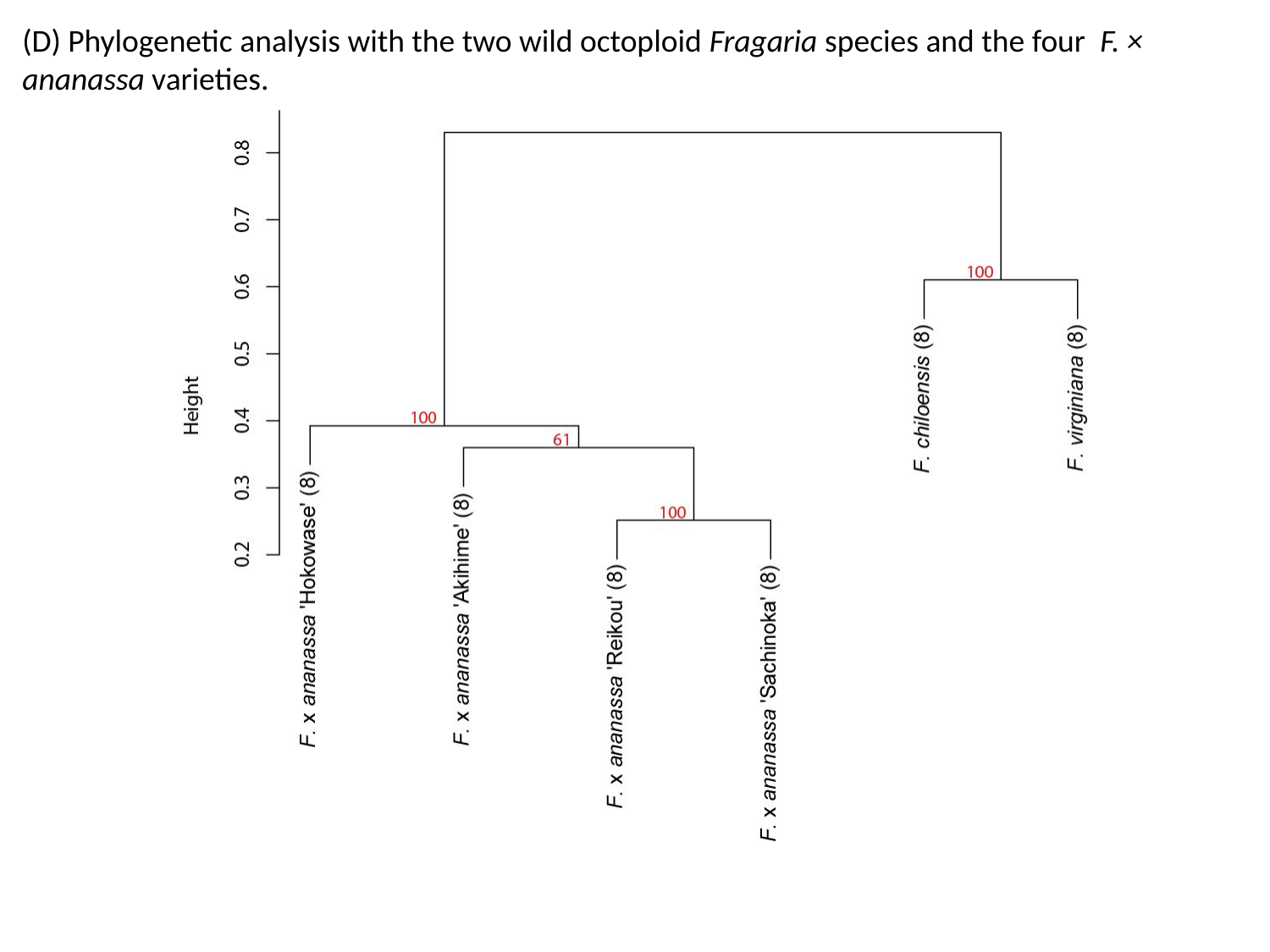

(D) Phylogenetic analysis with the two wild octoploid Fragaria species and the four F. × ananassa varieties.
