# Supplementary material for: Dissection of the Octoploid Strawberry Genome by Deep Sequencing of the Genomes of Fragaria Species
Source: DNA Res. 2013 Nov 26;21(2):169–81. doi: 10.1093/dnares/dst049 (PMC3989489; doi:10.1093/dnares/dst049)
Supplement: Supplementary Data [file supp_dst049_dst049supp_fig2.ppt]

## Slide 1
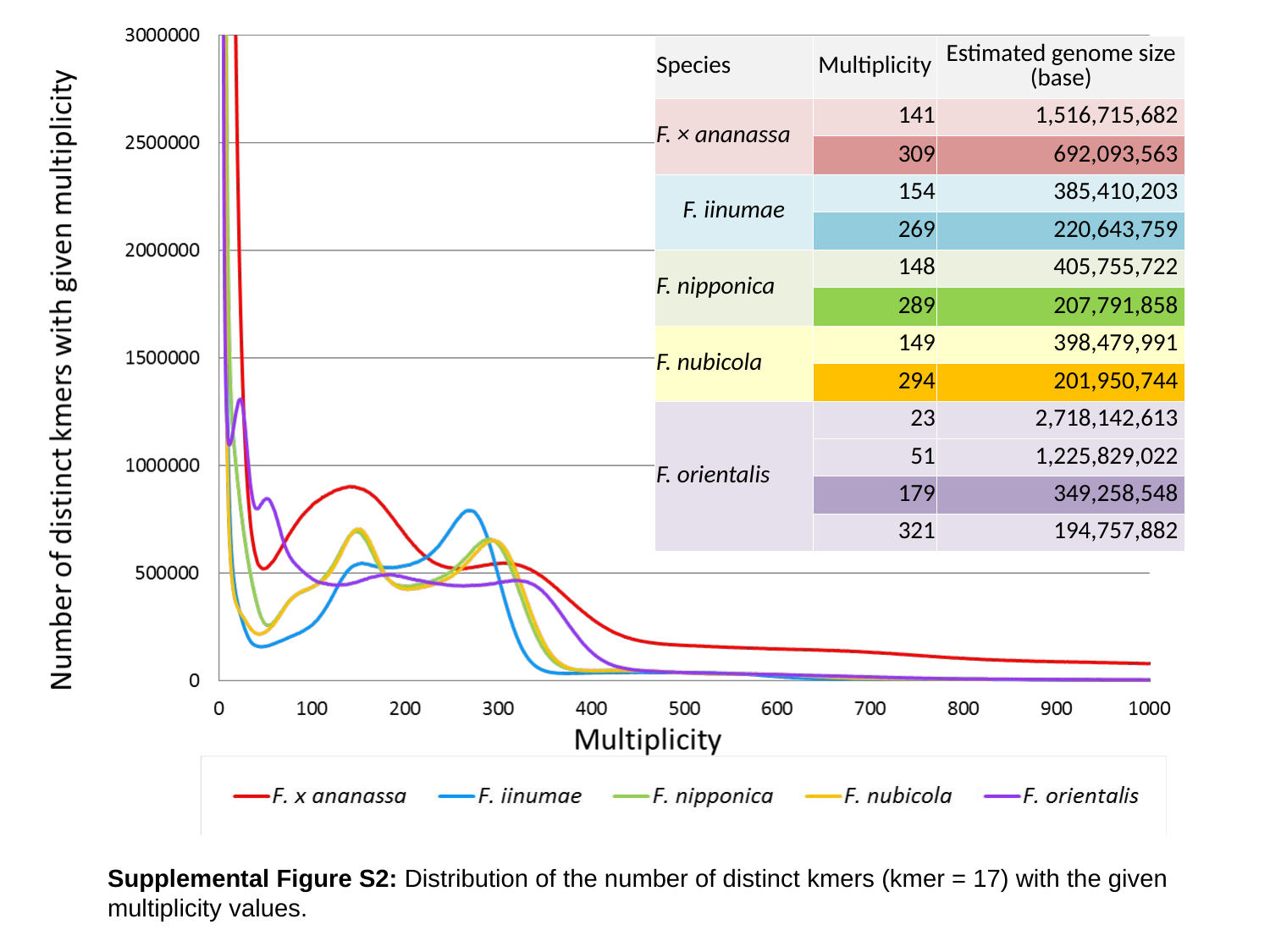

| Species | Multiplicity | Estimated genome size (base) |
| --- | --- | --- |
| F. × ananassa | 141 | 1,516,715,682 |
| | 309 | 692,093,563 |
| F. iinumae | 154 | 385,410,203 |
| | 269 | 220,643,759 |
| F. nipponica | 148 | 405,755,722 |
| | 289 | 207,791,858 |
| F. nubicola | 149 | 398,479,991 |
| | 294 | 201,950,744 |
| F. orientalis | 23 | 2,718,142,613 |
| | 51 | 1,225,829,022 |
| | 179 | 349,258,548 |
| | 321 | 194,757,882 |
Supplemental Figure S2: Distribution of the number of distinct kmers (kmer = 17) with the given multiplicity values.
