# Supplementary material for: Dissection of the Octoploid Strawberry Genome by Deep Sequencing of the Genomes of Fragaria Species
Source: DNA Res. 2013 Nov 26;21(2):169–81. doi: 10.1093/dnares/dst049 (PMC3989489; doi:10.1093/dnares/dst049)

## Slide 1
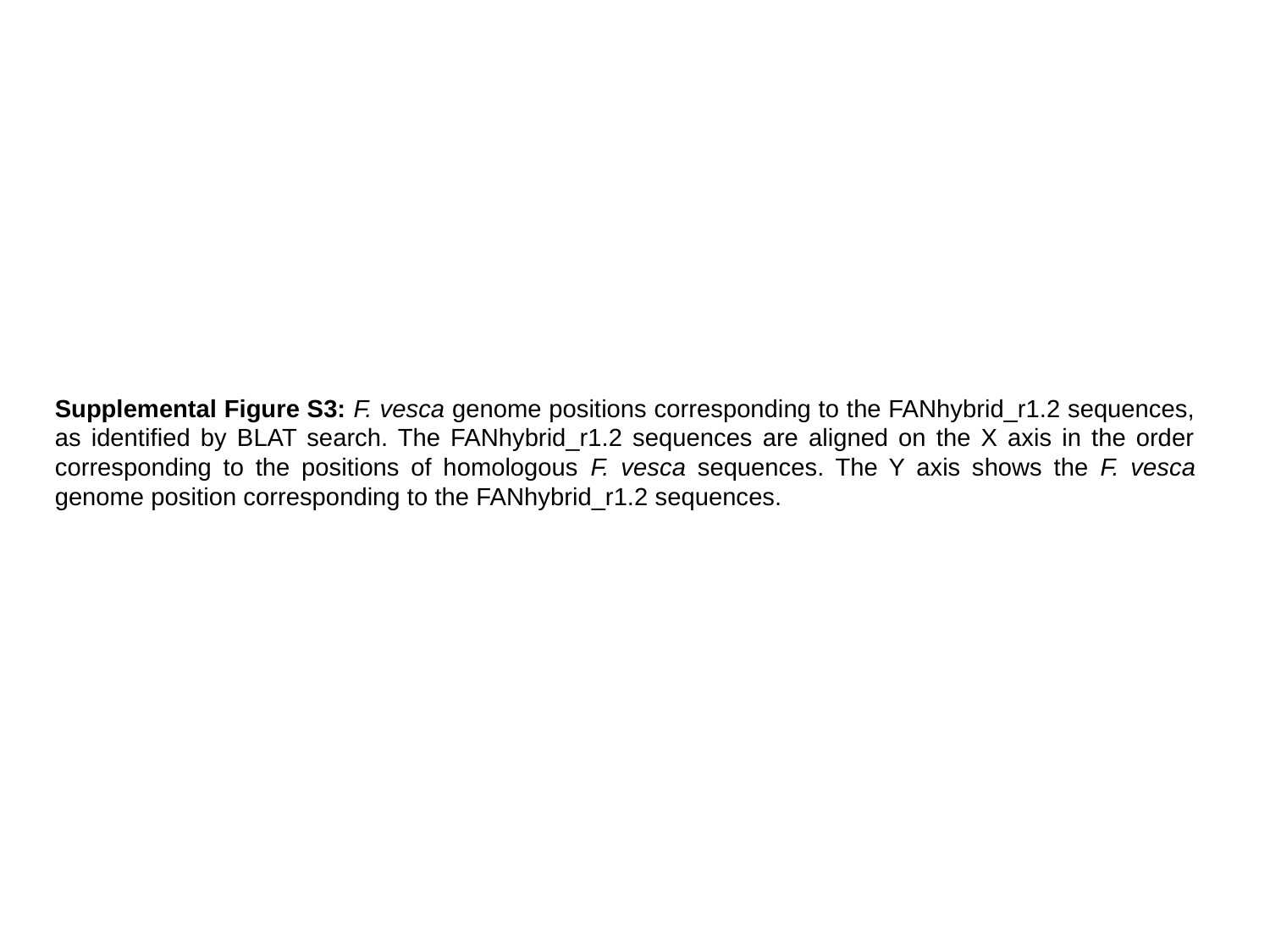

Supplemental Figure S3: F. vesca genome positions corresponding to the FANhybrid_r1.2 sequences, as identified by BLAT search. The FANhybrid_r1.2 sequences are aligned on the X axis in the order corresponding to the positions of homologous F. vesca sequences. The Y axis shows the F. vesca genome position corresponding to the FANhybrid_r1.2 sequences.

## Slide 2
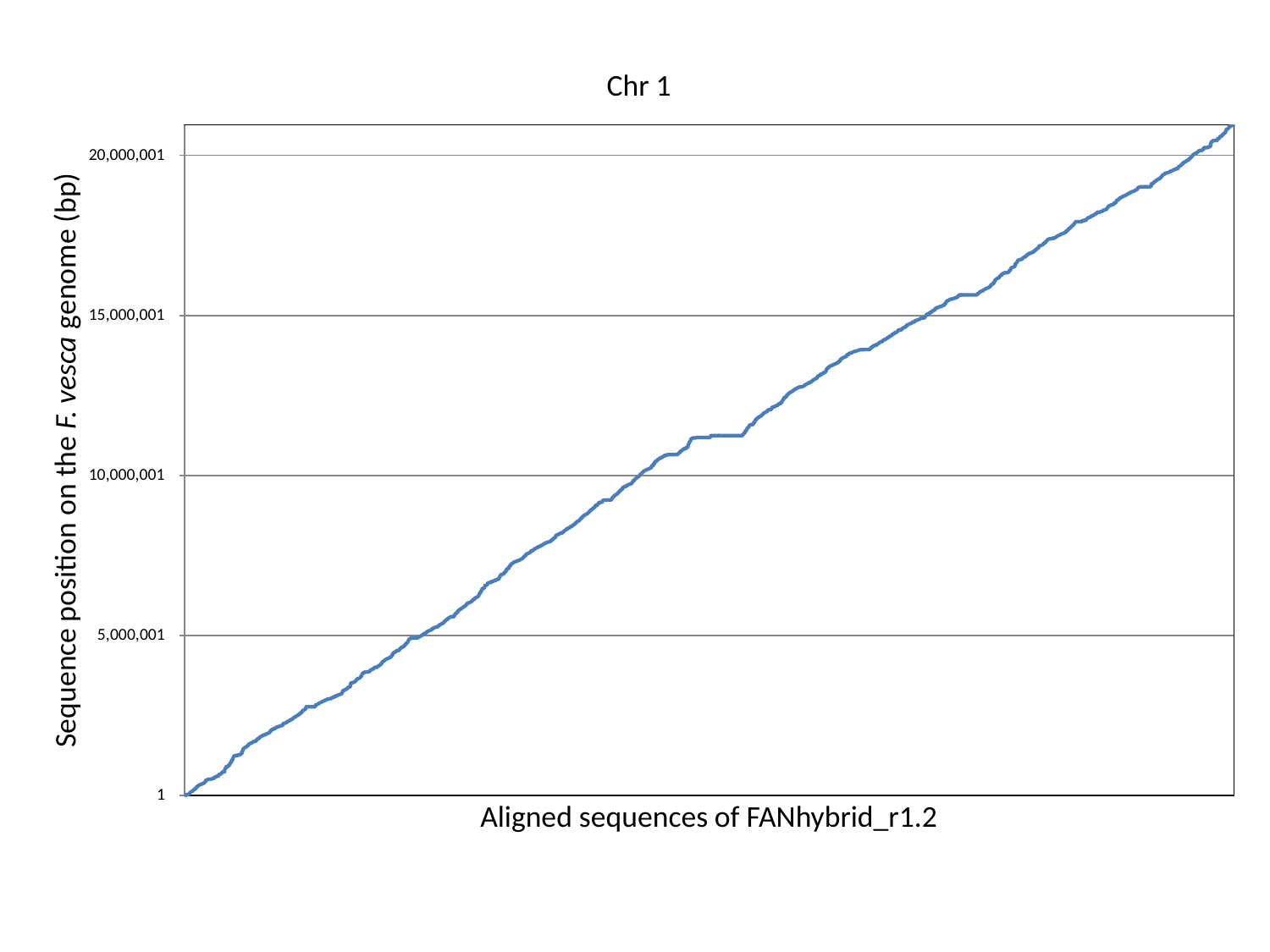

## Slide 3
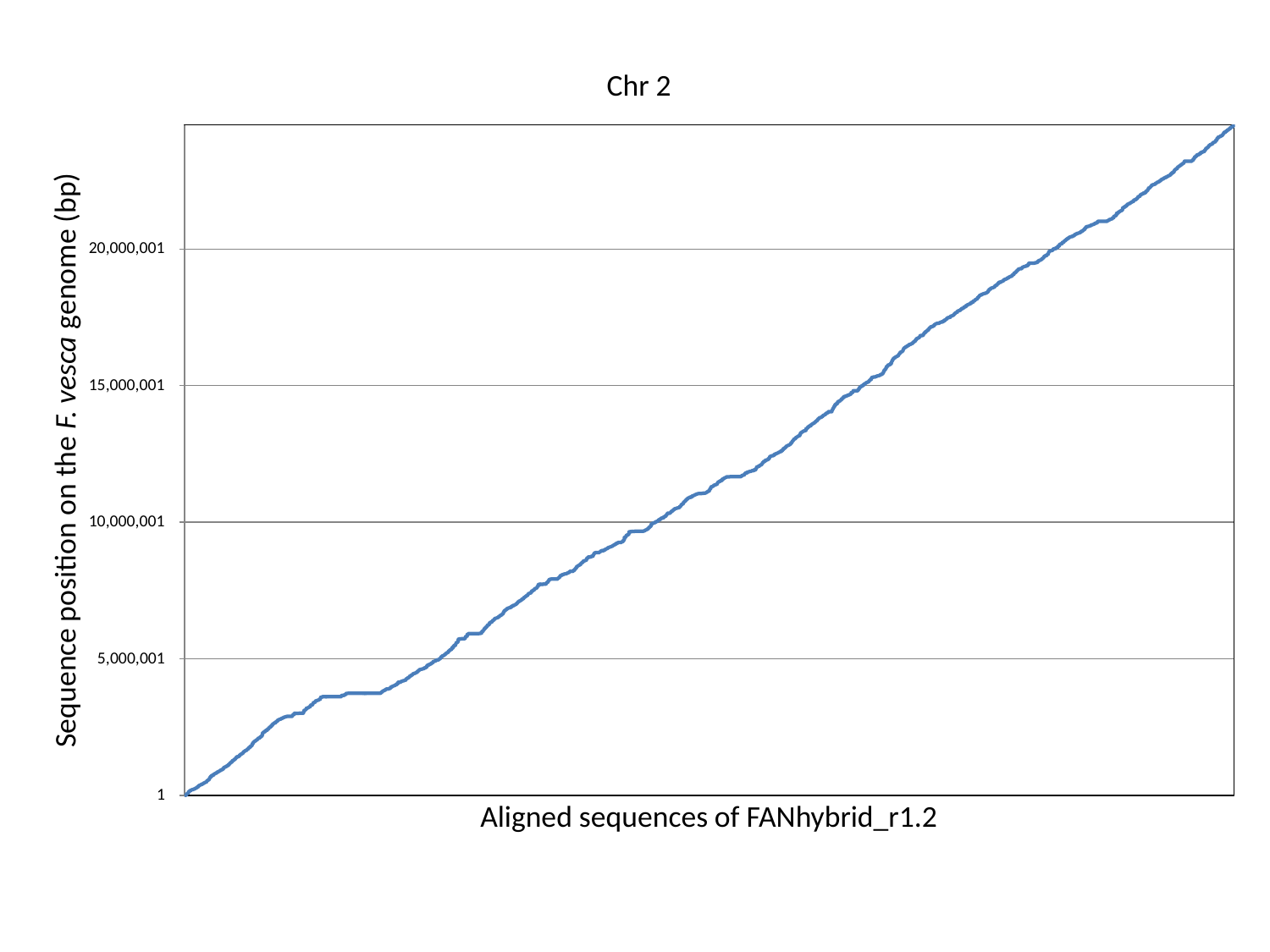

## Slide 4
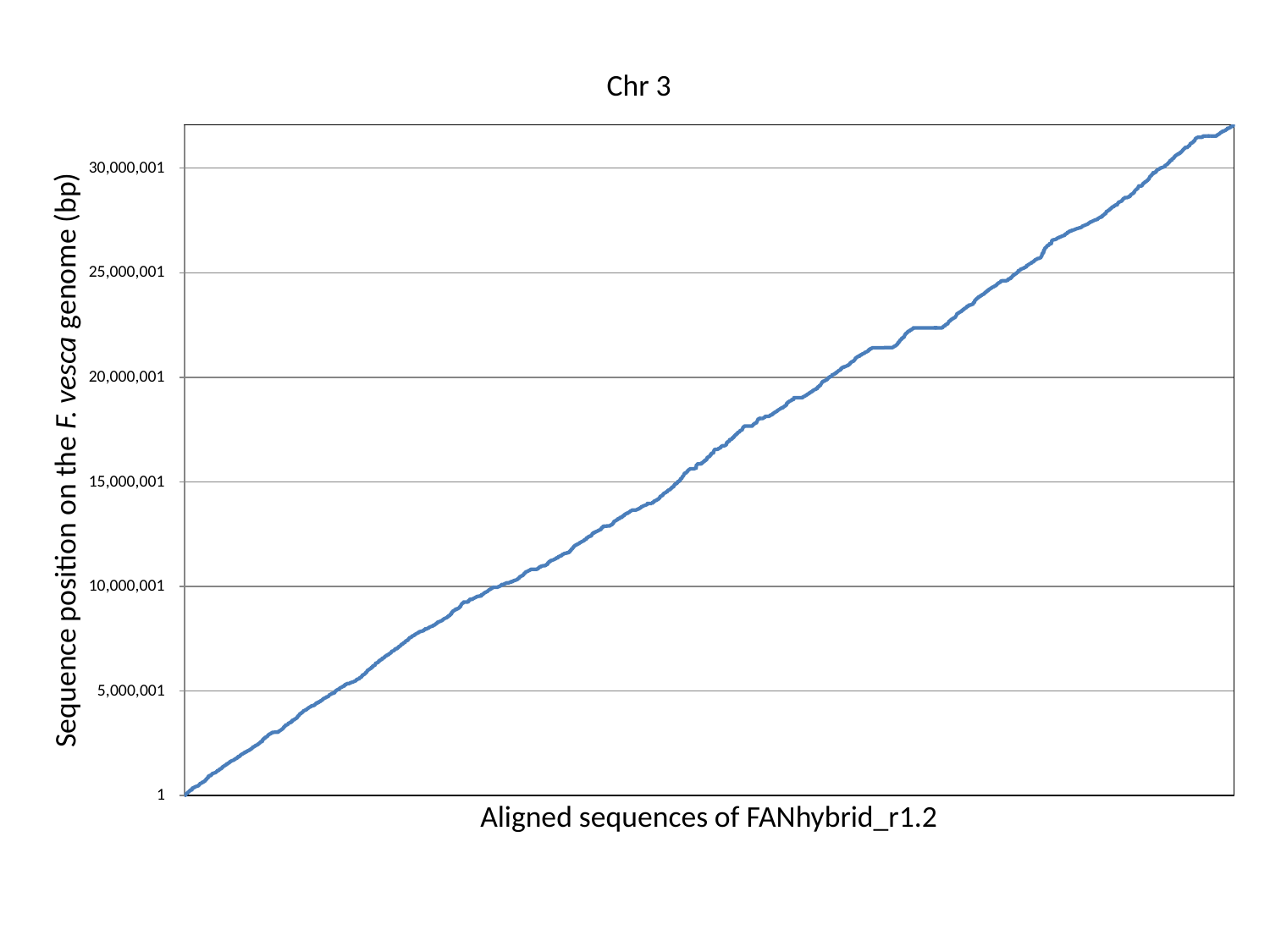

## Slide 5
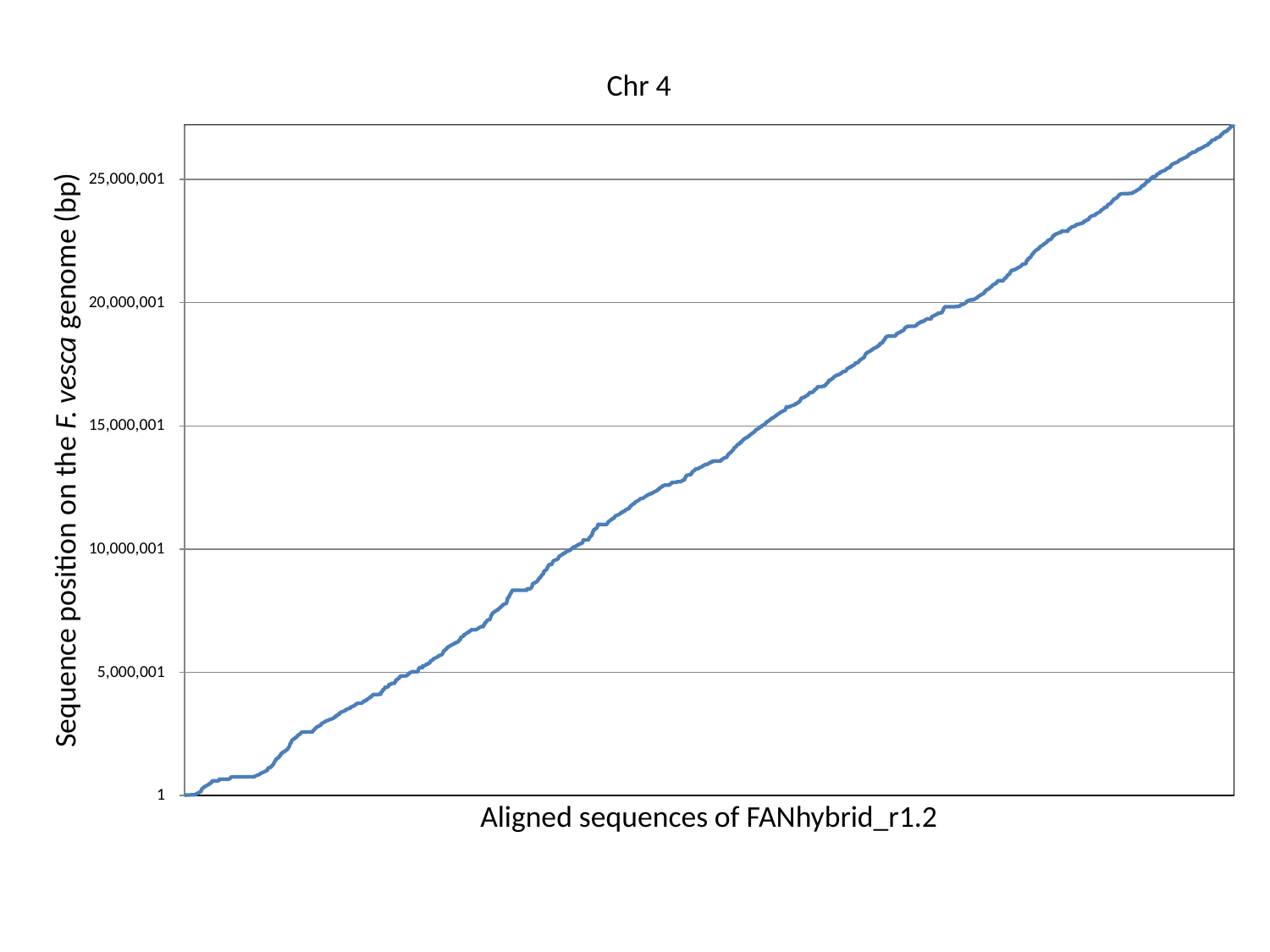

## Slide 6
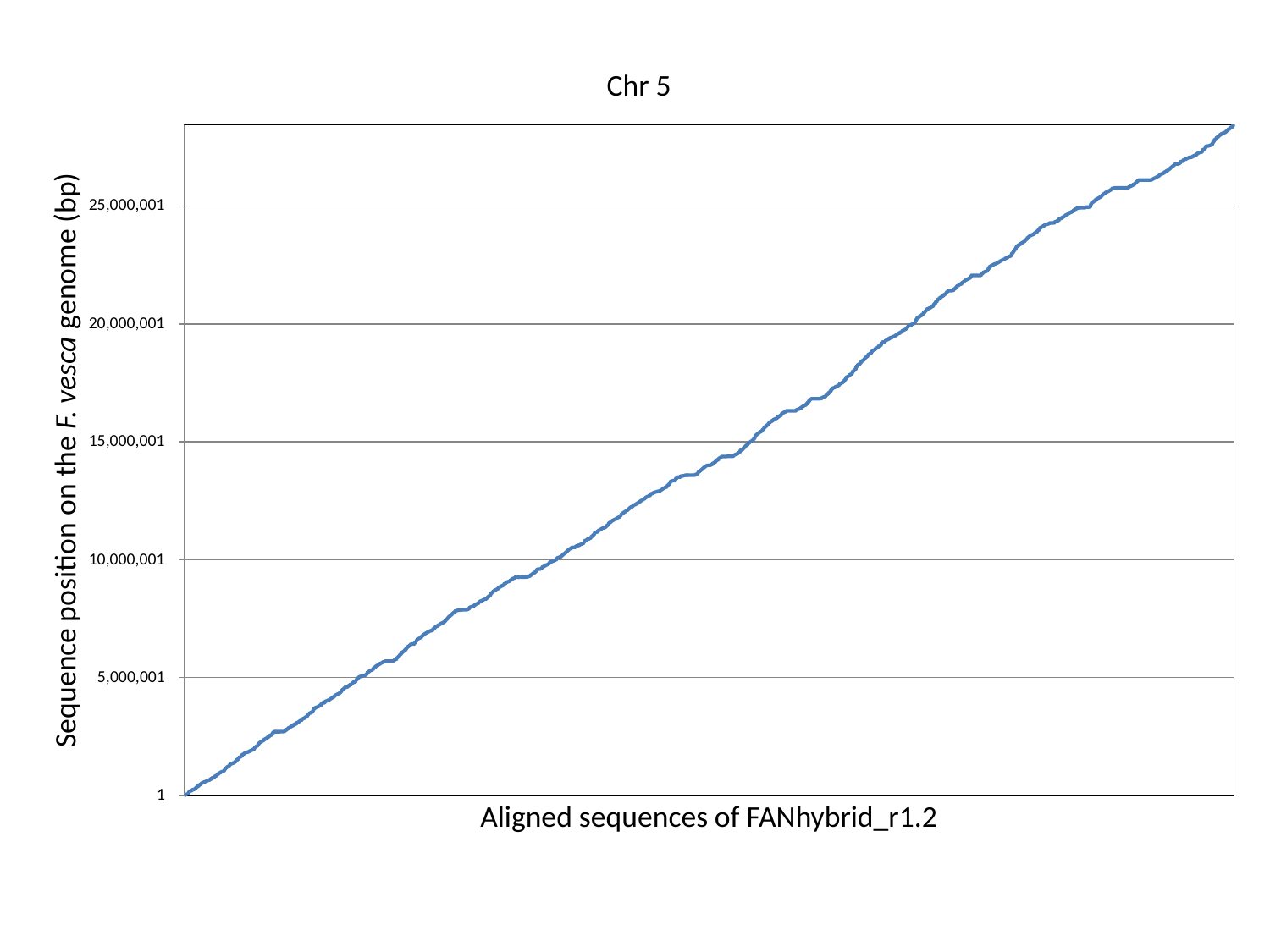

## Slide 7
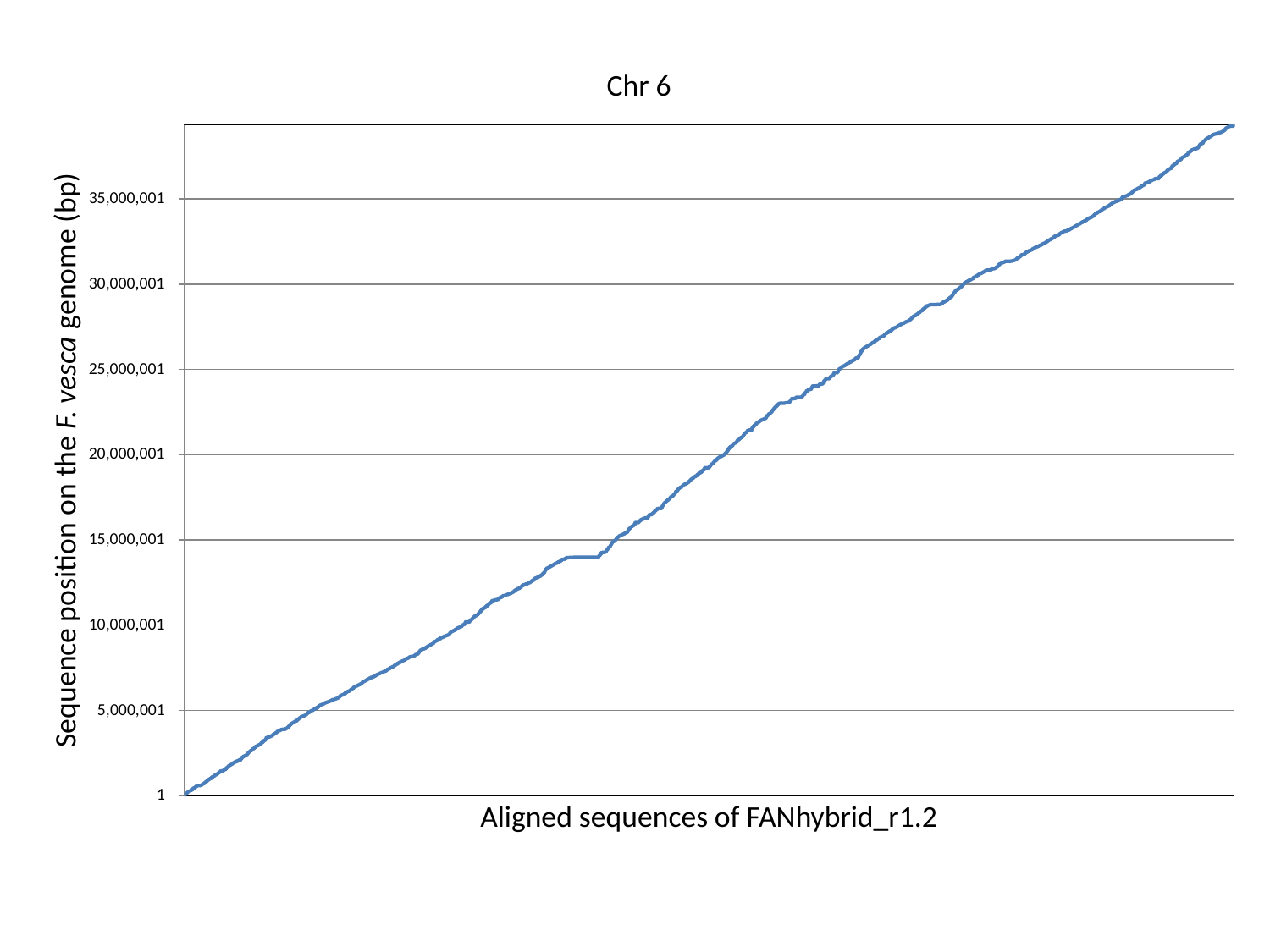

## Slide 8
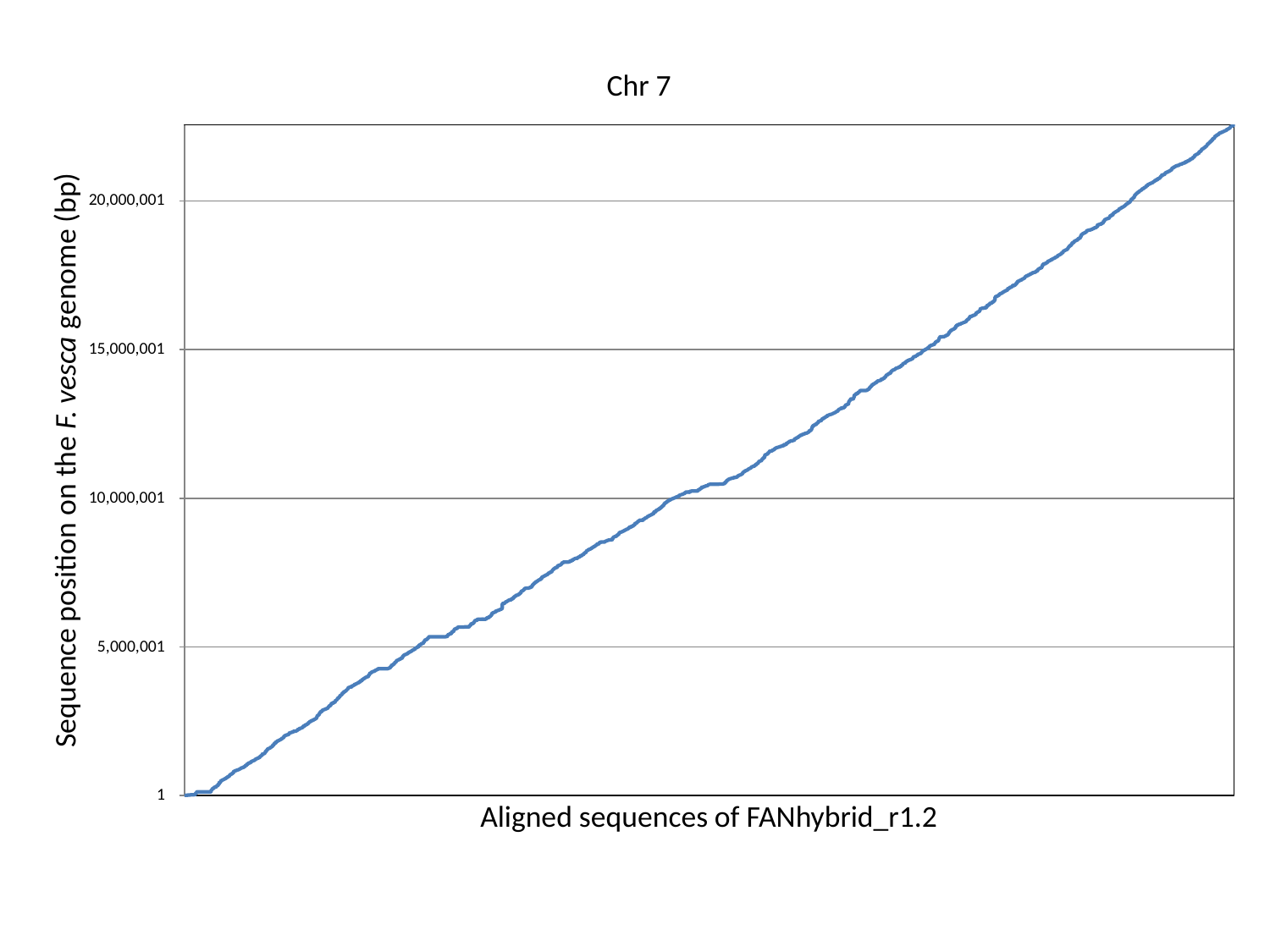

Supplement: Supplementary Data [file supp_dst049_dst049supp_fig3.ppt]
