# Supplementary material for: Dissection of the Octoploid Strawberry Genome by Deep Sequencing of the Genomes of Fragaria Species
Source: DNA Res. 2013 Nov 26;21(2):169–81. doi: 10.1093/dnares/dst049 (PMC3989489; doi:10.1093/dnares/dst049)
Supplement: Supplementary Data [file supp_dst049_dst049supp_fig6.ppt]

## Slide 1
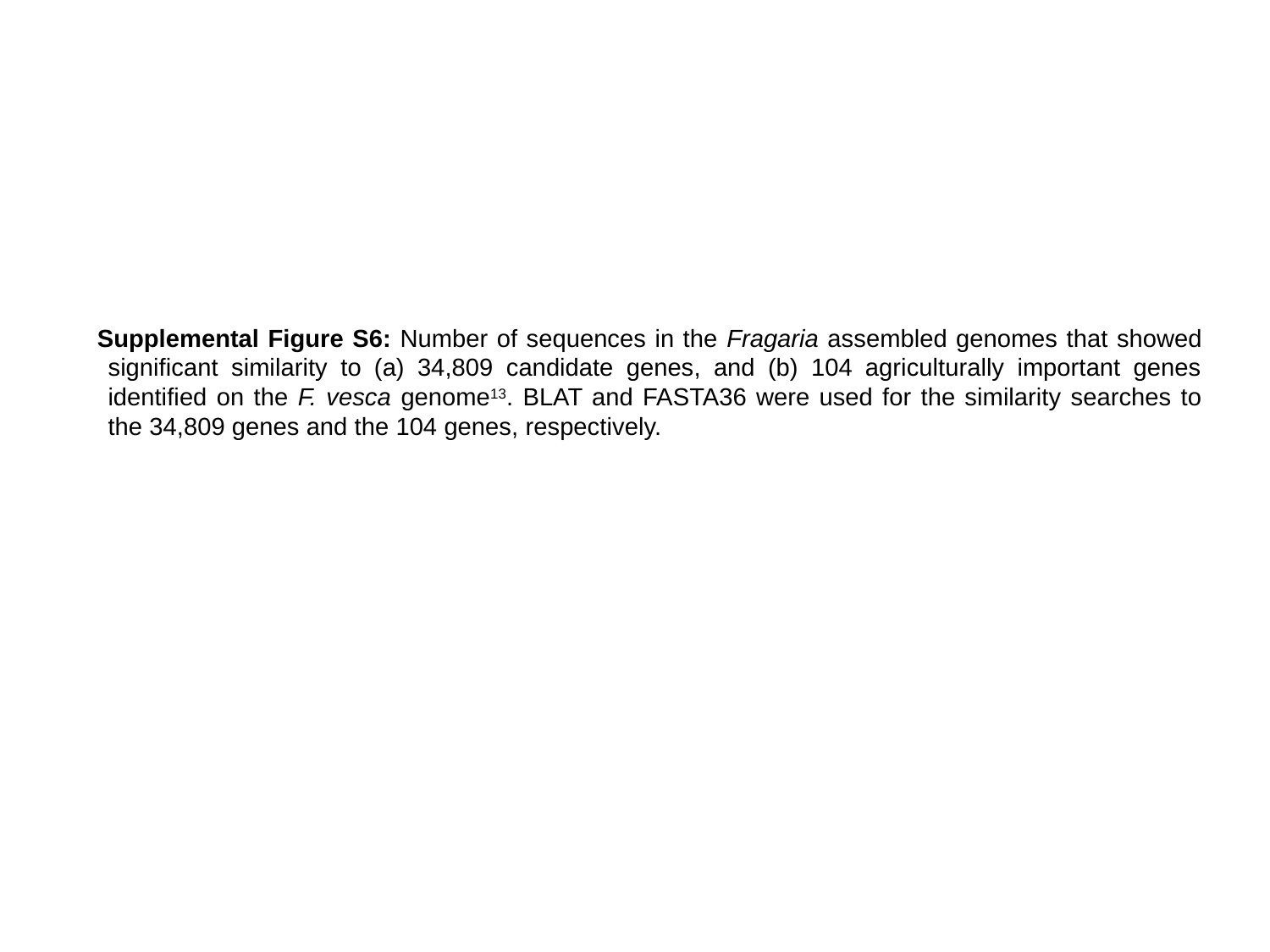

Supplemental Figure S6: Number of sequences in the Fragaria assembled genomes that showed significant similarity to (a) 34,809 candidate genes, and (b) 104 agriculturally important genes identified on the F. vesca genome13. BLAT and FASTA36 were used for the similarity searches to the 34,809 genes and the 104 genes, respectively.

## Slide 2
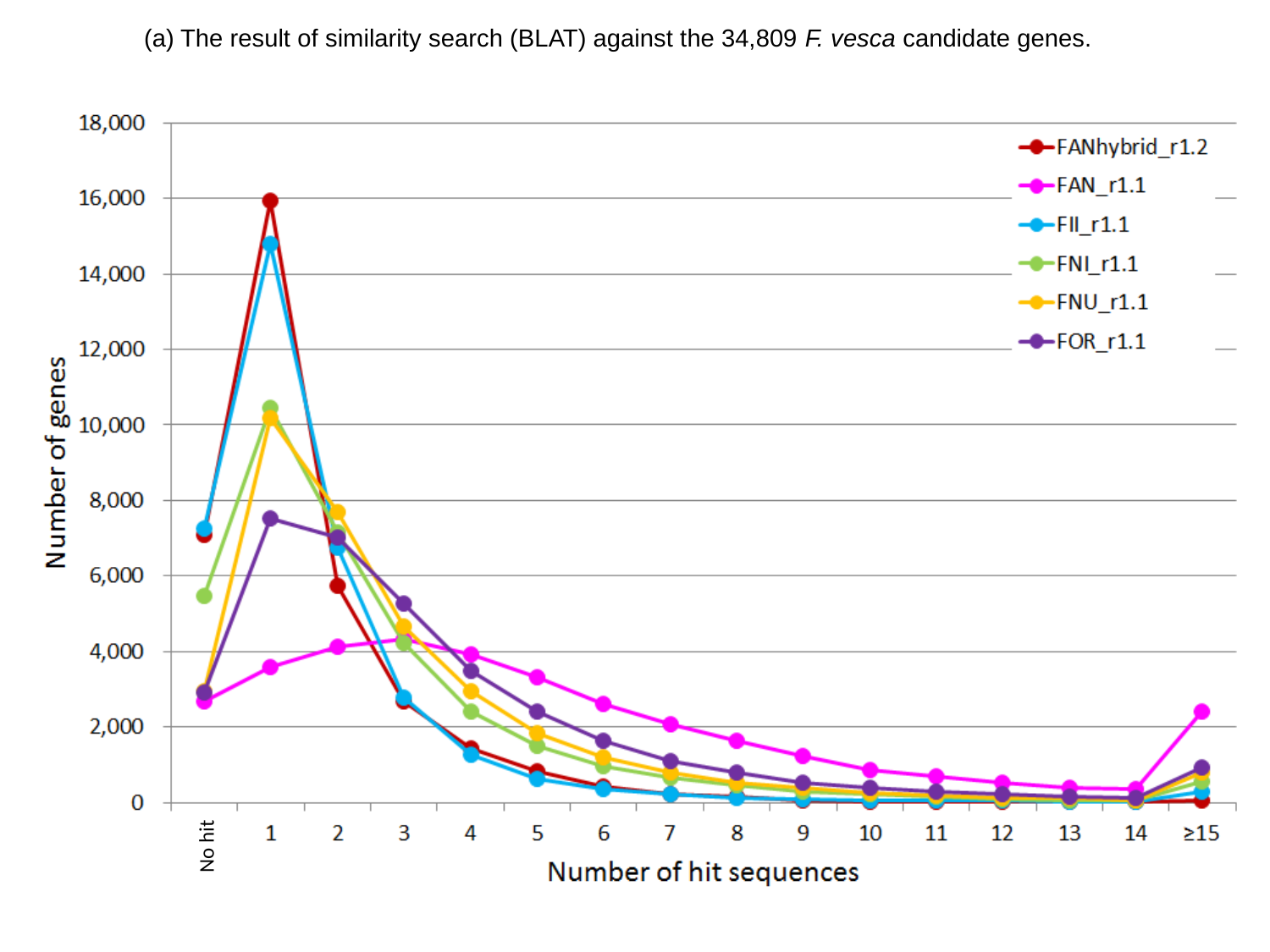

(a) The result of similarity search (BLAT) against the 34,809 F. vesca candidate genes.
No hit

## Slide 3
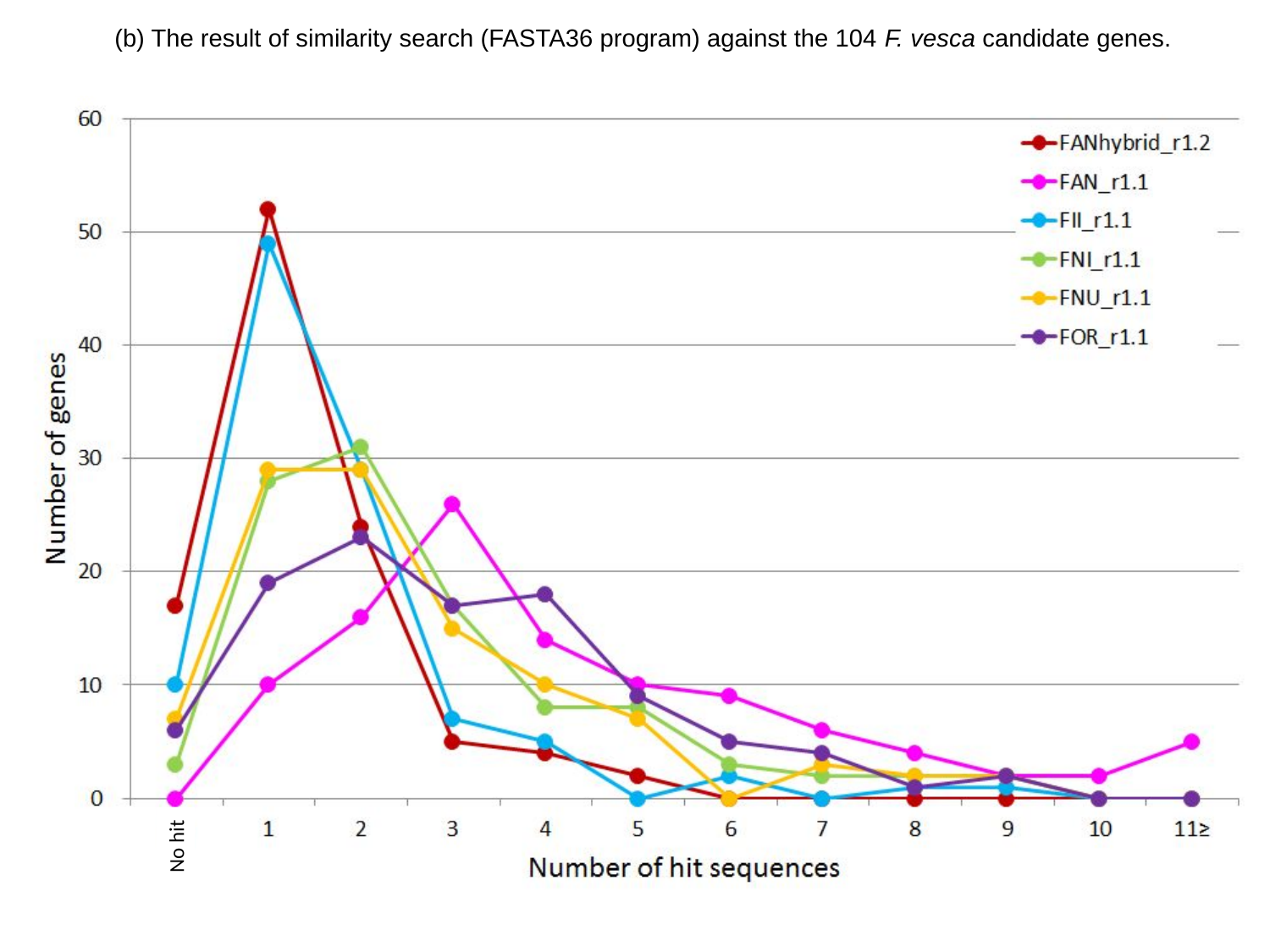

(b) The result of similarity search (FASTA36 program) against the 104 F. vesca candidate genes.
No hit
