# Supplementary material for: Dissection of the Octoploid Strawberry Genome by Deep Sequencing of the Genomes of Fragaria Species
Source: DNA Res. 2013 Nov 26;21(2):169–81. doi: 10.1093/dnares/dst049 (PMC3989489; doi:10.1093/dnares/dst049)
Supplement: Supplementary Data [file supp_dst049_dst049supp_fig7.ppt]

## Slide 1
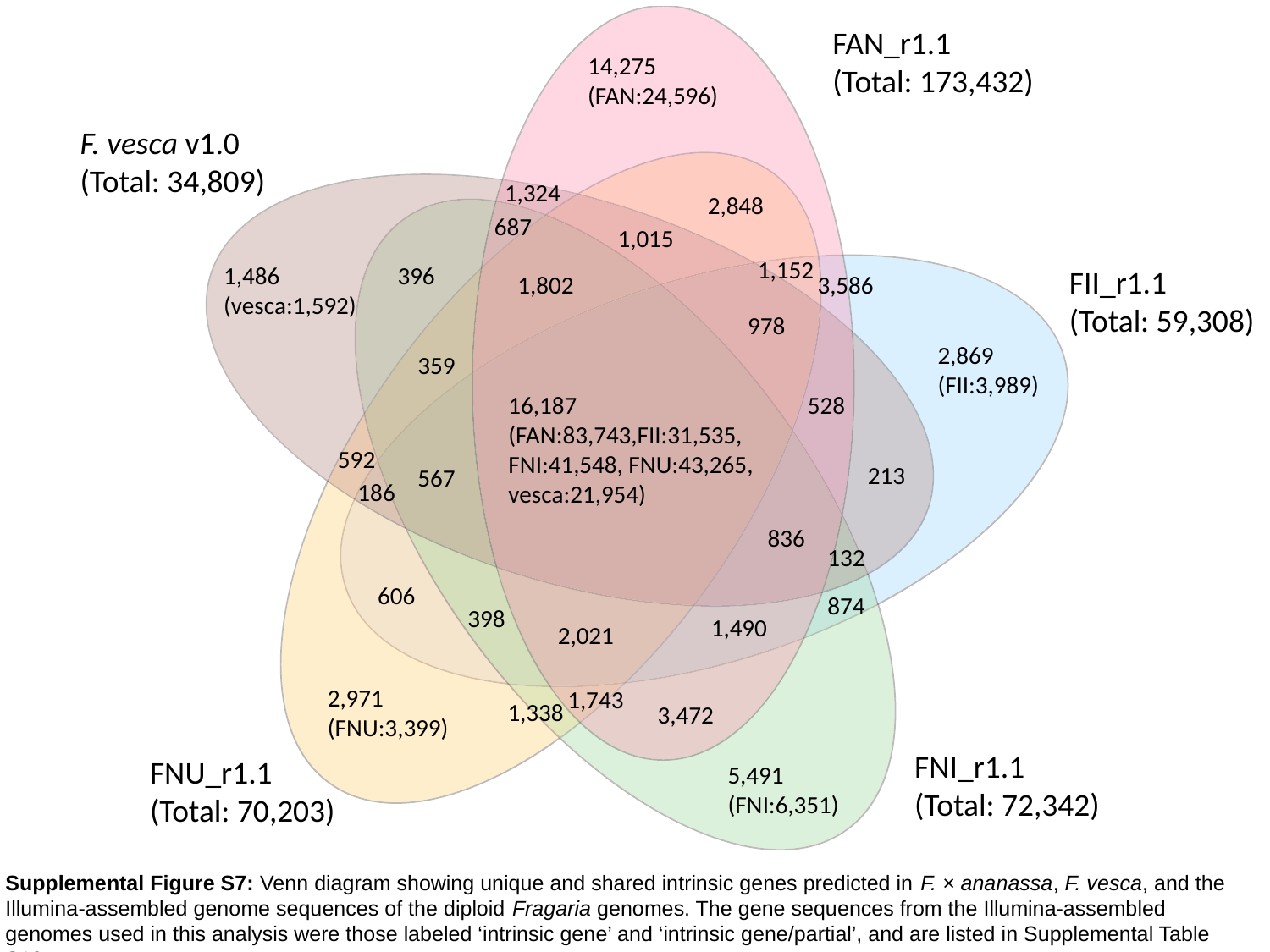

FAN_r1.1
(Total: 173,432)
14,275
(FAN:24,596)
F. vesca v1.0
(Total: 34,809)
1,324
2,848
687
1,015
1,152
1,486
(vesca:1,592)
396
FII_r1.1
(Total: 59,308)
1,802
3,586
978
2,869
(FII:3,989)
359
528
16,187
(FAN:83,743,FII:31,535, FNI:41,548, FNU:43,265, vesca:21,954)
592
213
567
186
836
132
606
874
398
1,490
2,021
2,971
(FNU:3,399)
1,743
1,338
3,472
FNI_r1.1
(Total: 72,342)
FNU_r1.1
(Total: 70,203)
5,491
(FNI:6,351)
Supplemental Figure S7: Venn diagram showing unique and shared intrinsic genes predicted in F. × ananassa, F. vesca, and the Illumina-assembled genome sequences of the diploid Fragaria genomes. The gene sequences from the Illumina-assembled genomes used in this analysis were those labeled ‘intrinsic gene’ and ‘intrinsic gene/partial’, and are listed in Supplemental Table S13.
